# Hyaluronic Acid Molecular Weight Modulates Chitosan–Gelatin Scaffold Properties and Cancer Cell Organization in 3D Culture

**DOI:** 10.3390/polym18141703

**Published:** 2026-07-10

**Authors:** Leimapokpam Romina Chanu, Guo-Chung Dong, Ping-Shan Lai

**Affiliations:** 1 Doctoral Program in Tissue Engineering and Regenerative Medicine, National Chung Hsing University, Taichung City 402204, Taiwan; romina.annauniversity@gmail.com; 2 Institute of Biomedical Engineering and Nanomedicine, National Health Research Institutes, Zhunan, Miaoli County 35053, Taiwan; 3 Department of Chemistry, National Chung Hsing University, Taichung City 402204, Taiwan

**Keywords:** hyaluronic acid, chitosan–gelatin scaffold, matrix biomechanics, cell–matrix interaction, 3D cancer models

## Abstract

Reconstructing physiologically relevant in vitro tumor microenvironments (TMEs) that capture coupled biophysical and biochemical complexities remains a significant challenge in cancer modeling. Here, we present a tunable chitosan–gelatin–hyaluronic acid (HA) scaffold platform stabilized by Schiff-base (C=N) crosslinking to investigate how HA molecular weight (MW) affects scaffold architecture, hydration behavior, mechanical properties, degradation stability, and cancer cell organization. We established an isocompositional series of HA-containing scaffolds, with HA MW as the primary variable and polymer composition held constant across all HA-containing groups. Varying HA MW modulated pore morphology, swelling behavior, compressive modulus, and degradation profiles, demonstrating distinct effects on scaffold structural organization. Medium-MW HA yielded a balanced scaffold architecture characterized by high porosity, controlled swelling behavior, and stable mechanical performance under hydrated conditions. A549 cells exhibited a compact, spheroid-like organization, whereas PANC-1 cells displayed more protrusive, spread morphologies that varied with scaffold formulation. Conversely, HA-free scaffolds showed reduced structural stability and less organized three-dimensional (3D) cell morphology. Collectively, these findings substantiate that HA MW is an effective design parameter for tuning scaffold physicochemical properties and influencing scaffold-associated cancer cell organization in 3D culture. This study provides a tunable scaffold platform for future in vitro tumor microenvironment modeling.

## 1. Introduction

The engineering of in vitro tumor microenvironments (TMEs) has shifted paradigmatically from simple cell aggregation toward the design of complex three-dimensional (3D) scaffolds that emulate the biophysical and biochemical heterogeneity of the native extracellular matrix (ECM). Within the native TME, the ECM functions not merely as a passive structural framework but also as an active regulator of cancer progression, in which spatially heterogeneous cues, such as matrix stiffness, pore architecture, and ligand availability, influence cancer cell organization, metastasis, and therapeutic resistance [1,2]. Consequently, the development of advanced in vitro tumor models requires material platforms capable of systematically tuning these biophysical parameters within a controlled 3D architecture [3]. This constitutes a fundamental materials science challenge in the development of biomimetic TME models, as existing scaffolding strategies often fail to decouple biophysical properties from biochemical composition, leading to confounding experimental variables that obscure the interpretation of cellular behavior in 3D tumor models [3].

Current strategies for TME reconstruction rely heavily on two distinct categories of materials, each exhibiting inherent limitations in isolating biophysical variables. Natural hydrogels, such as type I collagen or reconstituted basement membrane matrices (e.g., Matrigel), offer excellent bioactivity; however, they are constrained by unavoidable parameter coupling. In these systems, increasing matrix stiffness typically requires higher polymer concentrations, which in turn alter pore architecture and adhesive ligand density [4]. Consequently, cellular responses associated with matrix stiffness cannot be disentangled from those arising due to haptotaxis (ligand density sensing) or steric confinement (pore-scale architectural constraints) [5,6]. This coupling problem remains a fundamental obstacle, often leading to contradictory findings in cancer invasion and drug-response studies, as the effects of matrix density cannot be disentangled from those of matrix rigidity [4].

Conversely, semi-synthetic hydrogels, particularly methacrylated hyaluronic acid (HAMA) or gelatin (GelMA), have gained popularity for their photocrosslinking chemistries, which enable stiffness modulation independent of polymer concentration. While this approach provides enhanced physical tunability, it may compromise biological fidelity. Previous studies indicate that chemical modification of hyaluronic acid (HA) hydroxyl groups with bulky methacrylate moieties can sterically hinder or chemically disrupt CD44-binding epitopes, potentially affecting native HA–associated interactions [7,8]. Furthermore, radical-mediated photopolymerization has been reported to generate reactive oxygen species and oxidative stress [9], which may inadvertently alter cellular metabolic states or impose early selection pressures during the initial culture period. Thus, there is a critical need for material systems that preserve the native biochemistry of ECM components while enabling tunable physical architecture through intrinsic self-assembly and stabilized intermolecular interactions.

To address these limitations, we propose a design strategy that uses HA molecular weight (MW) as a primary design parameter in a tunable chitosan–gelatin–HA scaffold platform. By employing a polyelectrolyte complexation (PEC) framework subsequently stabilized by Schiff-base chemistry, the scaffold system was assembled through electrostatic interactions, chain entanglement, and hydration effects, and covalent stabilization [10,11,12]. An isocompositional series of HA-containing scaffolds was established using HA with low, medium, and high molecular weights while maintaining constant formulation and processing conditions across the HA-containing groups. This strategy enables modulation of physical architecture, including pore morphology, hydration behavior, swelling characteristics, and compressive properties, primarily through variation in HA chain length, without direct chemical modification of HA functional groups.

**Figure 1:**
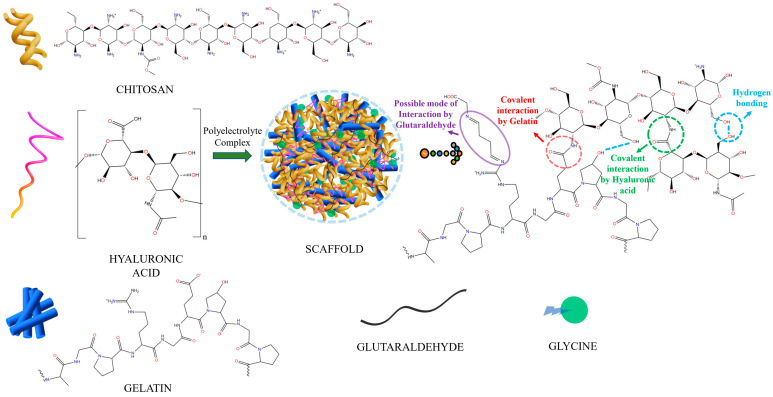
Conceptual schematic illustration of the fabrication process for chitosan–gelatin–hyaluronic acid (HA) scaffolds. The process involved: (1) polyelectrolyte complexation between cationic chitosan, amphoteric gelatin, and anionic HA of low (36 kDa), medium (180 kDa), and high (360 kDa) molecular weights; (2) Schiff base (–C=N–) crosslinking between glutaraldehyde and primary amine groups of chitosan/gelatin together with additional intermolecular physical interactions (e.g., hydrogen bonding and electrostatic attraction) within the polymer network; and (3) neutralization with glycine to quench unreacted aldehydes. All other formulation and processing parameters were held constant across the HA-containing scaffold groups, with HA molecular weight as the primary variable influencing the scaffold’s physicochemical organization.

We hypothesized that variation in HA molecular weight and scaffold physicochemical organization would influence scaffold-associated cancer cell organization within 3D culture, including compact spheroid-like organization in A549 cells and more protrusive cellular arrangements in PANC-1 cells. By preserving native ECM-associated chemistry while tuning the scaffold’s physical architecture through HA chain length, this work establishes a versatile scaffold platform for advanced studies of 3D tumor microenvironment and scaffold-associated cell organization (Figure 1).

## 2. Materials and Methods

### 2.1. Materials

Chitosan (low molecular weight, sourced from shrimp shells, degree of deacetylation ≥ 90.0%; GP8523) was procured from Glentham Life Sciences (Corsham, UK). Gelatin (Type A, derived from porcine skin, bloom strength of 300; G2500) and glacial acetic acid (ACS reagent, ≥99.7% purity; 695092) were acquired from Sigma-Aldrich (St. Louis, MO, USA). Glycine (intended for electrophoresis; G0317) and glutaraldehyde (~50% in water and ~5.6 mol/L; G0068) were obtained from Tokyo Chemical Industry (Tokyo, Japan). Isopropanol (≥99.5% purity; I0163) was purchased from Tasco Chemical Corporation (Taiwan). Dulbecco’s Phosphate Buffered Saline (DPBS, in powder form, adjusted to pH 7.4; 21600010), Dulbecco’s Modified Eagle’s Medium (DMEM; 12100046), Ham’s F-12K (Kaighn’s) Medium (21127022), and fetal bovine serum (FBS; A5670401) were obtained from Gibco™ (Thermo Fisher Scientific, Waltham, MA, USA). Hyaluronic acid (HA) samples with molecular weights of 36 kDa, 180 kDa, and 360 kDa, graciously supplied by Holy Stone Health Care (Taipei, Taiwan), were used as received. The human non-small cell lung carcinoma (A549) and pancreatic ductal adenocarcinoma (PANC-1) cell lines were obtained from the Bioresource Collection and Research Center (BCRC, Taiwan). Cell Counting Kit-8 (CCK-8; C0005) was purchased from TargetMol (Boston, MA, USA).

### 2.2. Scaffold Fabrication

Porous 3D scaffolds composed of chitosan, gelatin, and HA with varying molecular weights were fabricated to establish tunable biomimetic matrices. HA was incorporated into an isocompositional HA-containing scaffold series in three molecular-weight categories: low (LMW-HA, 36 kDa), medium (MMW-HA, 180 kDa), and high (HMW-HA, 360 kDa), using the same operational definitions throughout the study. The concentrations of chitosan (1–3% *w*/*v*), gelatin (1–10% *w*/*v*), and HA (0.1–2% *w*/*v*) were carefully selected from ranges widely used in the tissue-engineering literature [13,14,15] to ensure relevance and biocompatibility. Detailed formulations of each scaffold, including specific concentration values and respective nomenclature, are presented in Table 1. Briefly, an HA-free chitosan–gelatin scaffold was prepared as the control group, whereas an isocompositional HA-containing scaffold series was prepared by incorporating HA of different molecular weights while maintaining constant chitosan, gelatin, and HA concentrations among the HA-containing groups. Chitosan, gelatin, and the corresponding HA types were dissolved in 1% (*v*/*v*) acetic acid under continuous stirring at 50 °C for 2 h to obtain homogeneous polymer solutions. The physical appearance of the polymer solutions and the resulting macroscopic morphology of the lyophilized scaffolds are provided in the Supplementary Materials (Figures S1 and S2).

The polymer solutions were cast onto circular molds and frozen at −80 °C overnight. The resulting freeze-cast scaffold discs were sectioned into cylindrical discs of the desired thickness using a biopsy punch. The discs were subsequently lyophilized for 48 h to generate porous 3D scaffolds. Crosslinking was performed by immersing the scaffolds in 1% (*v*/*v*) glutaraldehyde for 4–5 h, followed by neutralization with 1.5% (*w*/*v*) glycine and extensive washing with Milli-Q water to reduce the residual unreacted aldehyde groups. The scaffolds were then refrozen, lyophilized under the same conditions, and stored in a desiccator until further use. A conceptual schematic illustration of the overall fabrication process is presented in Figure 1.

### 2.3. Surface Morphology and Microstructural Analysis (SEM)

The surface morphology and microstructure of the freeze-dried scaffolds were examined using a scanning electron microscope (SEM; HITACHI S-3400N; Hitachi, Tokyo, Japan) operated at 15 kV. Cell-free (blank) scaffolds were imaged to assess pore architecture, surface texture, and interconnectivity. For the biological assessment, cell-seeded scaffolds were collected on days 1, 3, and 6 post-seeding from both A549 and PANC-1 cultures to examine cell attachment, spreading, and scaffold infiltration. Before imaging, all samples were sputter-coated with a thin layer of gold to enhance surface electrical conductivity and minimize charging artifacts. Quantitative pore-size measurements were performed on blank scaffolds using ImageJ 1.54d (National Institutes of Health, USA). Measurements were obtained from multiple regions across independent samples (*n* = 3) to ensure a representative and statistically reliable analysis.

### 2.4. Chemical Functional Group Analysis by FT-IR Spectroscopy

Fourier-transform infrared (FT-IR) spectroscopy analysis was performed to investigate the chemical composition and potential intermolecular interactions within the scaffolds. Lyophilized scaffolds were analyzed in both uncrosslinked and crosslinked formulations using a Thermo Nicolet 6700 FT-IR spectrometer (Thermo Fisher Scientific, Madison, WI, USA) equipped with an attenuated total reflectance (ATR) accessory and a mercury cadmium telluride (MCT) detector. All spectra were recorded over the range of 650–4000 cm^−1^, with 16 scans per spectrum at a resolution of 4 cm^−1^. The acquired spectra were subsequently analyzed to identify characteristic absorption bands and assess chemical interactions associated with polyelectrolyte complexation and Schiff-base crosslinking.

### 2.5. Swelling Behavior

The swelling behavior of the freeze-dried scaffolds was evaluated to quantify their hydration capacity under physiological conditions. Pre-weighed dry samples were immersed in Dulbecco’s phosphate-buffered saline (DPBS, pH 7.4) and incubated at 37 °C. At predetermined time points (Days 1, 3, 6, 9, 15, 25, and 30), the scaffolds were carefully removed, rinsed three times with sterile double-distilled water to eliminate residual salts, gently blotted to remove surface moisture, and immediately weighed to obtain the wet weight (W_w_). Subsequently, the samples were lyophilized, and the final dry weight (W_f_) was recorded. Swelling measurements were performed in triplicate for each formulation (*n* = 3). The swelling ratio (%) was calculated using Equation (1), as reported previously [16]:

(1) $$Swelling\;ratio\left(\%\right)=\;\frac{W_{w}-W_{f}}{W_{f}}\times 100$$

### 2.6. Pore Size Quantification and Microarchitectural Analysis

Quantitative pore-size analysis was performed using SEM images of blank, freeze-dried scaffolds. Images captured at appropriate magnifications were analyzed using ImageJ 1.54d software (National Institutes of Health, USA). For each scaffold formulation, at least 30 randomly selected pores were measured per sample, with three independent samples analyzed (*n* = 3). The mean pore diameter was calculated and reported as mean ± standard deviation.

### 2.7. Evaluation of Total Porosity

The total porosity of the scaffolds was evaluated using a modified liquid-displacement method, as described previously [11,17]. Isopropanol, a non-solvent, served as the displacement liquid owing to its capability to penetrate the porous structure without causing scaffold swelling or degradation. Briefly, dried scaffolds were weighed to obtain the initial mass (W_i_) and subsequently immersed in isopropanol of known density (ρ_i_) for 30 min to ensure complete infiltration. Following immersion, the isopropanol-saturated scaffolds were retrieved, gently blotted to remove excess surface liquid, and reweighed (W_f_). The porosity (%) was calculated using the following formula:

(2) $$Porosity\left(\%\right)=\;\frac{(W_{f}-W_{i}){/\rho }_{i}}{V_{i}}\times 100$$

where V_i_ is the initial geometric volume of the dry scaffold, calculated from its measured dimensions.

All measurements were conducted using 12 replicates per scaffold formulation. Precautions were taken to ensure thorough saturation of the scaffold with isopropanol, thereby preventing any air entrapment within the porous network.

### 2.8. Mechanical Properties Characterization

The compressive mechanical properties of the scaffolds were evaluated using a RapidTA texture analyzer (R-TA, Horn Instruments Co., Ltd., Taoyuan, Taiwan) in accordance with ASTM D1621-16 standards [18], with minor modifications to accommodate the scaffolds’ geometry. Scaffolds from all four formulations were tested under three distinct conditions: dry (freeze-dried), wet (DPBS-equilibrated), and cell-laden.

Compressive testing in the dry state was performed immediately after freeze-drying to establish the baseline mechanical properties of the inherent polymeric network. For the cell-laden conditions, A549 and PANC-1 cells were seeded onto the scaffolds at a density of 1 × 10^6^ cells per scaffold, as determined from preliminary optimization studies to ensure uniform cell distribution and spheroid formation. Cells were seeded in 50 µL of complete culture medium, allowed to adhere for 2 h, and then transferred to fresh medium. Subsequently, the cell-seeded scaffolds were cultured at 37 °C in a humidified atmosphere containing 5% CO_2_ and harvested at 1, 3, and 6 days for mechanical testing immediately after removal from the incubator.

Under wet conditions, scaffolds were incubated in DPBS (pH 7.4) at 37 °C for the same time intervals as in cell culture. Before testing, DPBS-equilibrated scaffolds were carefully blotted to remove excess surface fluid. DPBS provides a physiological ionic strength that promotes charge shielding of electrostatic interactions between the polyelectrolyte components (chitosan and HA) [19,20], thereby modulating swelling behavior and yielding mechanical properties that more closely reflect those of in vitro culture environments. This parallel experimental design enabled a direct comparison between hydrated and cell-laden scaffolds under physiologically relevant conditions.

All samples were tested as cylindrical specimens. Cell-seeded scaffolds of formulations CH–G and CH–G–HA360 measured 6 mm in diameter and 3 mm in height, whereas formulations CH–G–HA36 and CH–G–HA180 had dimensions of 5 mm in diameter and 2 mm in height. All dry and DPBS-equilibrated scaffolds were prepared with a uniform diameter of 5 mm and a height of 2 mm. Uniaxial compression tests were conducted at room temperature with a crosshead speed of 0.05 mm s^−1^ until a strain of 50% was achieved. The compressive modulus of all samples was calculated from the linear region (typically 10–20% strain) of the stress–strain curves, excluding the initial toe region, to focus on the elastic response of the scaffold architecture. Stress (*σ*) was defined as the applied force normalized to the initial cross-sectional area (*σ* = *F*/*A*), whereas strain (*ε*) was defined as the change in displacement normalized to the original scaffold height (*ε* = Δ*L*/*L*_0_). Representative stress–strain curves are provided in Supplementary Materials Figures S4–S6. Fold-change values were calculated as the ratio between the compressive modulus of cell-laden scaffolds and the modulus of corresponding acellular hydrated scaffolds. All measurements were performed in triplicate (*n* = 3) for each formulation, and the data are presented as the mean ± standard deviation.

### 2.9. In Vitro Hydrolytic Degradation and Structural Stability

The in vitro hydrolytic degradation behavior of the scaffolds was evaluated over 30 days under physiological conditions. Pre-weighed, freeze-dried scaffolds (W_i_) were immersed in sterile DPBS (pH 7.4) and incubated at 37 °C under static conditions. At predetermined intervals (Days 1, 3, 6, 9, 15, 25, and 30), samples were retrieved, gently washed with sterile double-distilled water to remove residual soluble salts, and subsequently freeze-dried to obtain the final dry weight (W_f_). The percentage of mass loss (degradation) was calculated from weight loss at each time point using Equation (3), adapted from previously reported methods [19].

(3) $$Degradation\left(\%\right)=\frac{\left(W_{i}-W_{f}\right)}{W_{i}}\times 100$$

All degradation measurements were performed in triplicate for each formulation at each time point, and the results were expressed as mean ± standard deviation.

### 2.10. Cell Lines and Culture Conditions

Human lung carcinoma (A549) and pancreatic carcinoma (PANC-1) cell lines were used to evaluate the scaffold’s cytocompatibility and cancer cell–specific responses. A549 cells were cultured in Ham’s F-12K Medium, while PANC-1 cells were maintained in DMEM. Both culture media were supplemented with 10% FBS and 1% penicillin–streptomycin–neomycin (PSN). Cells were incubated under standard conditions in a humidified atmosphere at 37 °C with 5% CO_2_ and passaged when approximately 80% confluent.

Prior to cell seeding, the scaffolds were sterilized by ultraviolet (UV) irradiation for 30 min and subsequently pre-equilibrated in complete culture medium for a few hours to facilitate cell attachment. For all biological assays, cells were seeded onto the sterile scaffolds at a density of 1 × 10^6^ cells per scaffold, as determined from a preliminary optimization study. Seeding was performed in 50 µL of complete medium, with cells allowed to adhere for 2 h before adding fresh culture medium. All experiments were conducted over culture durations of 1, 3, and 6 days, selected to capture early attachment, intermediate organization, and later-stage three-dimensional cell assembly.

### 2.11. Metabolic Activity (CCK-8 Assay)

Relative metabolic activity of A549 and PANC-1 cells cultured on the scaffolds was evaluated using the CCK-8 assay in accordance with the manufacturer’s instructions. Briefly, on days 1, 3, and 6 post-seeding, the culture medium was replaced with fresh medium containing 10% (*v*/*v*) CCK-8 reagent, and the cultures were then incubated at 37 °C for 3 h. Following this, 100 µL of the reacted solution from each well was transferred to a 96-well plate, and absorbance was measured at 450 nm using a Multiskan SkyHigh microplate spectrophotometer (Thermo Fisher Scientific, Waltham, MA, USA). Background subtraction was performed using a complete culture medium incubated with 10% CCK-8 reagent as the liquid blank. To evaluate temporal changes in metabolic activity within each scaffold formulation, blank-subtracted absorbance values obtained on days 3 and 6 were normalized to the corresponding Day 1 value of the same experimental group, which was set at 100%. The data are presented as the mean ± standard deviation (SD), with five biological replicates per formulation at each time point (*n* = 5). The experiments were repeated independently three times for each cell type to confirm reproducibility.

### 2.12. Cancer Cells Morphodynamics and Scaffold Interactions (SEM)

Scanning electron microscopy (SEM) analysis was conducted to examine cancer cell attachment, morphology, and interactions with the scaffold matrix. Scaffold specimens seeded with A549 and PANC-1 cells were collected at days 1, 3, and 6 post-seeding. At each designated time point, the scaffolds were carefully rinsed with DPBS (pH 7.4) and fixed in 10% formaldehyde at room temperature (22–25 °C) for approximately 16 h to ensure complete fixation throughout the three-dimensional structure and optimal preservation of cellular morphology. Subsequently, the fixed samples were dehydrated through a graded ethanol series (30%, 50%, 70%, 80%, 90%, 95%, and 100%), with each step lasting for 10–15 min. The specimens were then subjected to critical-point drying, sputter-coated with a thin layer of gold, and examined using a SEM (HITACHI S-3400N; Hitachi, Tokyo, Japan) operated at 15 kV. Cell morphology, spatial organization, and interactions with the scaffold architecture were qualitatively evaluated.

### 2.13. Statistical Analysis

All quantitative experiments were conducted with at least three independent replicates (*n* ≥ 3) per experimental group, unless otherwise specified in the respective sections. The number of replicates was selected to evaluate relative differences among scaffold formulations in the exploratory characterization experiments performed in this study. Data are presented as mean ± standard deviation (SD). Statistical comparisons among multiple groups were performed using one-way analysis of variance (ANOVA), followed by Tukey’s honestly significant difference (HSD) post hoc test. Statistical analyses were conducted using OriginPro 2018 (OriginLab Corporation, Northampton, MA, USA). A *p*-value of less than 0.05 was considered statistically significant. In the figures, statistically significant differences are indicated by asterisks (* *p* < 0.05). Due to the exploratory nature of certain scaffold characterization experiments, statistical interpretations were performed cautiously and supported by consistent experimental trends and complementary characterization results.

## 3. Results

### 3.1. Scaffold Design and Characterization Rationale

The chitosan–gelatin–HA scaffolds were engineered as hierarchical dual-network systems in which physical polyelectrolyte complexation (PEC) and covalent Schiff-base crosslinking synergistically produce mechanically stable yet tunable 3D architectures (Figure 1). Upon dissolution in acetic acid, chitosan is protonated, whereas gelatin carries a net positive charge, enabling electrostatic interactions with the anionic carboxylate groups of HA and promoting the formation of an initial PEC network [12,20]. HA molecular weight (MW) influences PEC formation by modulating chain flexibility, charge accessibility, and entanglement density, thereby affecting polymer packing, pore formation during lyophilization, and hydration-associated behavior [21]. Chitosan and gelatin were chosen as complementary structural components to balance mechanical integrity and biological functionality within the scaffold system. Chitosan contributes structural reinforcement and electrostatic interactions within the PEC network [22,23], whereas gelatin provides integrin-binding motifs that promote cell adhesion [24,25]. HA was incorporated as an ECM-associated component known to influence hydration behavior, viscoelastic characteristics, and degradation profiles, with HA MW serving as a primary design parameter to modulate scaffold architecture and compressive properties while maintaining a constant polymer composition across the HA-containing scaffold series [26]. Covalent stabilization was subsequently introduced through glutaraldehyde-mediated Schiff-base (C=N) linkages between aldehydes and the primary amine groups of chitosan and gelatin, followed by glycine neutralization to reduce residual aldehyde-associated cytotoxicity [27,28]. This crosslinking strategy reinforced the PEC network while preserving the polymer system’s overall physicochemical characteristics.

Importantly, HA contains no primary amines and therefore does not participate directly in Schiff-base crosslinking. Instead, its incorporation occurs predominantly through physical interactions, including electrostatic interactions, hydrogen bonding, and chain entanglement within the crosslinked chitosan–gelatin matrix [29,30,31]. To investigate the influence of HA chain length on the scaffold’s physicochemical organization, HA was incorporated at low (36 kDa), medium (180 kDa), and high (360 kDa) MWs, while maintaining constant formulation composition, crosslinking conditions, and processing parameters across the HA-containing scaffold series. Under these strictly controlled and isocompositional HA-containing conditions, variations in pore morphology, swelling behavior, degradation kinetics, and compressive response were interpreted in relation to MW-associated differences in PEC organization, hydrogen bonding, and physical entanglement. This design rationale established the foundation for evaluating subsequent scaffold–associated cell organization and morphology within a unified material system.

### 3.2. Chemical Characterization and Network Formation (FT-IR Analysis)

**Figure 2:**
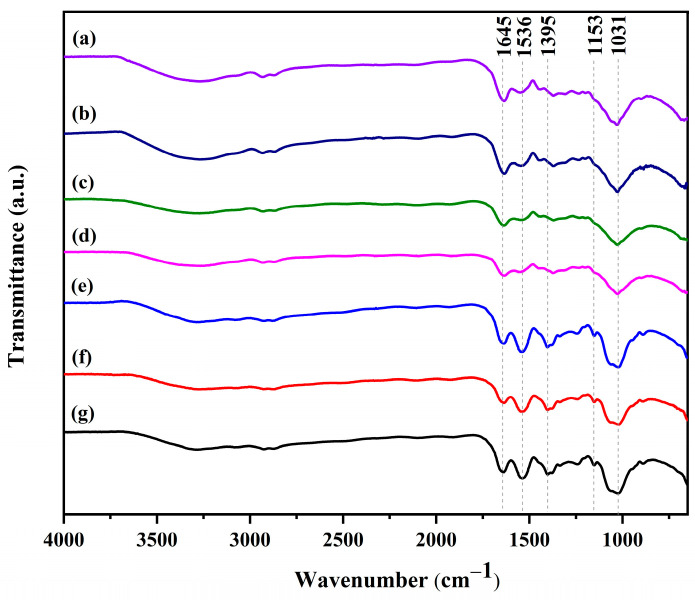
FT-IR spectra of the scaffold system showing spectral features associated with scaffold crosslinking and polyelectrolyte complexation. Spectra have been vertically offset for clarity. The trace assignments, as detailed in Table 1, are as follows: (a) crosslinked CH–G (control); (b) crosslinked CH–G–HA360; (c) crosslinked CH–G–HA180; (d) crosslinked CH–G–HA36; (e–g) the corresponding uncrosslinked formulations. Vertical dashed lines indicate characteristic spectral regions near 1641 cm^−1^ (amide I/C=O region with possible imine contributions), 1536 cm^−1^ (amide II region), 1395 cm^−1^ (–COO^−^-associated region), 1153 cm^−1^ (C–O–C-associated region), and 1031 cm^−1^ (C–O stretching of the polysaccharide backbone).

FT-IR spectroscopy was employed to evaluate chemical interactions and network formation within the chitosan–gelatin–HA scaffolds (Figure 2). The spectra of uncrosslinked mixtures (Figure 2) exhibited characteristic features associated with the three polymeric components, including a broad band at 3307 cm^−1^ attributable to O–H and N–H stretching vibrations [32,33], amide I (1641 cm^−1^) and amide II (1536 cm^−1^) bands primarily associated with gelatin with overlapping contributions from chitosan and HA [32,34,35], a prominent band at 1031 cm^−1^ corresponding to C–O stretching of the polysaccharide backbone [35,36], a shoulder near 1153 cm^−1^ associated with C–O–C stretching in glycosidic linkages [37], and a band at 1395 cm^−1^ corresponding to the symmetric stretching of HA carboxylate groups (–COO^−^) [38,39].

Following glutaraldehyde crosslinking (Figure 2), several spectral changes consistent with network formation were observed. The reduced prominence of the 1153 cm^−1^ C–O–C-associated band suggests an alteration in the local glycosidic environment following scaffold stabilization. In addition, decreased intensity of the amide II band near 1536 cm^−1^ is consistent with partial consumption of primary amine groups during crosslinking, while subtle shifts and intensity variations within the 1641 cm^−1^ region reflect overlapping contributions from amide I (C=O) vibrations and possible imine (C=N) formation [32]. Together, these spectral changes are consistent with Schiff-base-mediated network stabilization within the scaffold system. The reduction in intensity of the 1395 cm^−1^ –COO^−^ band following crosslinking is also consistent with electrostatic interactions between HA and chitosan within the PEC network and reduced HA mobility within the stabilized matrix [40,41]. Conversely, the 1031 cm^−1^ C–O-associated band remained comparatively preserved across the scaffold groups, suggesting retention of the polysaccharide backbone structure. Although the overall FT-IR profiles remained broadly similar across scaffolds containing varying HA molecular weights (Figure 2), subtle variations in band width and relative intensity, particularly near 1395 cm^−1^ and 1153 cm^−1^, suggest MW-associated differences in ionic interactions and local network organization. These observations align with the proposed scaffold design in which HA MW primarily influences physicochemical organization of the polymer network, while the dominant covalent crosslinking chemistry remains similar across the HA-containing scaffold series.

Collectively, the FT-IR results support the successful integration of chitosan, gelatin, and HA within the scaffold system and are consistent with PEC-associated interactions and Schiff-base-mediated stabilization. These findings provide a chemical basis for scaffold design and identify HA molecular weight as a key parameter that influences subsequent structural, mechanical, and biological observations.

### 3.3. Surface Morphological and Microstructural Analysis (SEM)

**Figure 3:**
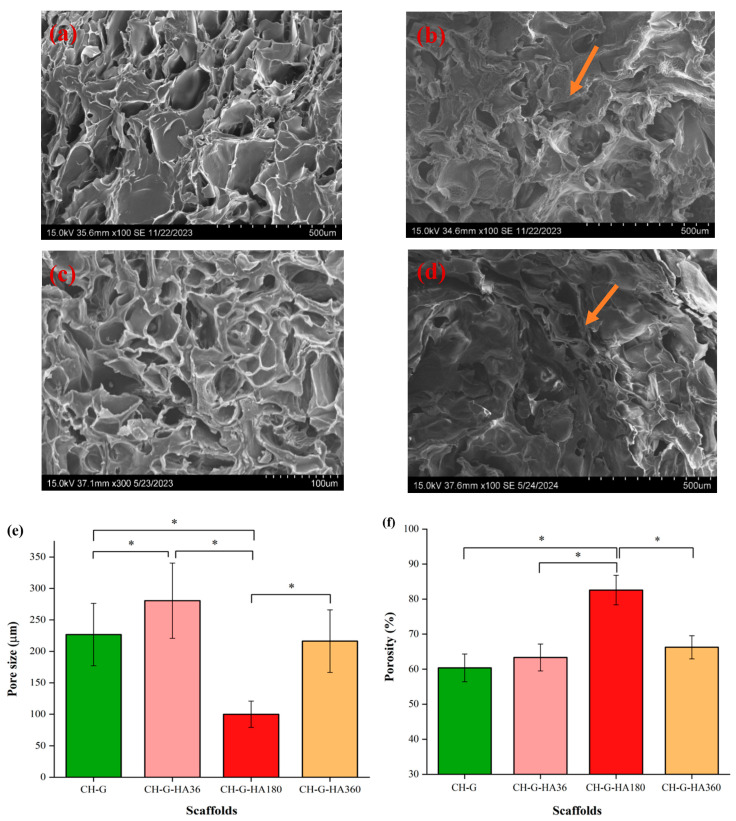
Morphological and architectural characteristics of the fabricated scaffolds. Representative SEM images of (**a**) CH–G (control), (**b**) CH–G–HA36 (LMW-HA), (**c**) CH–G–HA180 (MMW-HA), and (**d**) CH–G–HA360 (HMW-HA). Yellow arrows in images (**b**,**d**) indicate observed irregular pore morphology and localized structural heterogeneity, respectively. The comparatively finer, more uniform architecture of CH–G–HA180 (panel (**c**)) is shown at a higher magnification (scale bar: 100 µm) relative to panels (**a**,**b**,**d**) (scale bar: 500 µm). Quantitative analysis of (**e**) pore size and (**f**) porosity illustrates the observed morphological trends. Data are presented as mean ± SD (*n* = 3 for pore size and *n* = 12 for porosity). Statistical significance was evaluated using one-way ANOVA followed by Tukey’s post hoc test (* *p* < 0.05).

SEM was employed to evaluate the pore architecture, interconnectivity, and surface morphology of chitosan–gelatin scaffolds incorporating HA of varying molecular weights (Figure 3a–d). These architectural features influence nutrient transport, cell distribution, and scaffold-associated cellular organization in 3D culture systems [42,43], while also providing insight into the effects of HA molecular weight on scaffold assembly and pore formation during lyophilization. SEM micrographs revealed differences in scaffold morphology that were dependent on HA molecular weight. The HA-free control scaffold (CH–G) exhibited irregular, non-uniform pore structures with comparatively limited architectural organization (Figure 3a). Incorporation of LMW-HA (CH–G–HA36) resulted in surfaces with rough textures and irregular, partially fused pores (Figure 3b). Conversely, MMW-HA (CH–G–HA180) generated a relatively homogeneous and interconnected porous network with well-defined pore boundaries (Figure 3c), suggesting a more uniform polymer organization within the scaffold matrix. HMW-HA (CH–G–HA360) exhibited heterogeneous regions characterized by fused pores and irregular morphological features (Figure 3d), consistent with localized variations in network packing and pore stabilization associated with increased HA chain length.

### 3.4. Quantitative Analysis of Scaffold Pore Size

Quantitative pore-size analysis supported the qualitative SEM observations (Table 2, Figure 3e): CH–G–HA36 exhibited the largest mean pore size (280.6 µm), consistent with the observed pore fusion and irregular architecture. CH–G showed the second-largest pores (226.7 µm), reflecting the comparatively less organized pore structure of the HA-free chitosan–gelatin scaffold. CH–G–HA360 exhibited an intermediate mean pore size (216.2 µm), suggesting partial architectural heterogeneity within the scaffold network. Notably, CH–G–HA180 exhibited the smallest mean pore size (~100 µm) while maintaining a highly interconnected porous architecture. This pore-size range has previously been associated with favorable cell infiltration and multicellular organization in 3D scaffold systems [44,45]. Collectively, these observations suggest that medium-molecular-weight HA promoted comparatively balanced scaffold organization during lyophilization, whereas lower and higher HA molecular weights were associated with more irregular pore morphologies.

### 3.5. Porosity Analysis of Scaffolds

The scaffolds’ porosity demonstrated dependence on HA molecular weight (Figure 3f, Table 2). CH–G–HA180 exhibited the highest porosity (83%), followed by CH–G–HA360 (66%), CH–G–HA36 (63%), and CH–G (60%). The high porosity observed in CH–G–HA180, despite its comparatively smaller mean pore size, suggests that medium-molecular-weight HA promoted the formation of a relatively continuous and interconnected porous network. Conversely, the larger but more irregular pore structures observed in CH–G–HA36 may have reduced overall void uniformity due to localized pore collapse and structural heterogeneity, resulting in lower overall porosity. Importantly, the porosity of CH–G–HA180, exceeded the commonly cited threshold of ≥70% associated with efficient nutrient transport and waste exchange in 3D scaffold systems [46,47], supporting its functional suitability for scaffold-associated multicellular organization under prolonged culture conditions.

### 3.6. Mechanical Properties Under Dry, Hydrated, and Cell-Laden Conditions

**Figure 4:**
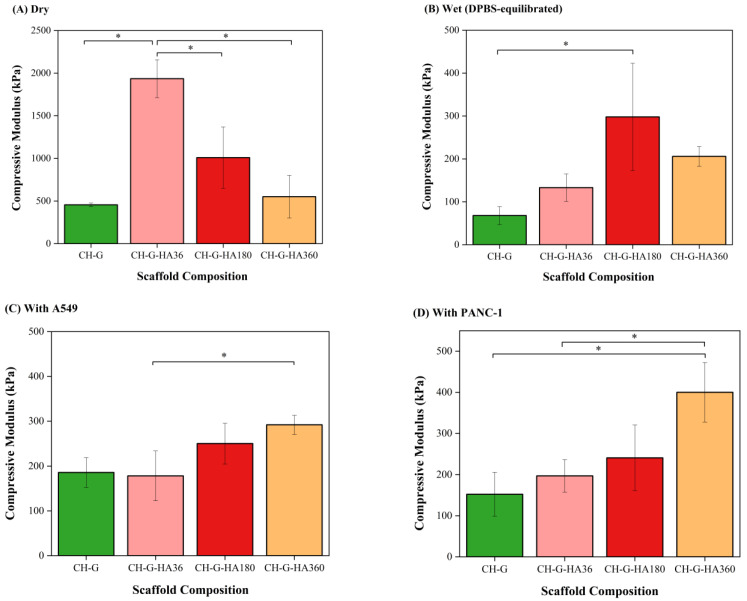
Compressive modulus of the scaffolds under various conditions: (**A**) Dry, (**B**) Wet (DPBS-equilibrated for 6 days), (**C**) A549 cell-laden (6 days), and (**D**) PANC-1 cell-laden (6 days). Variations in HA molecular weight and culture condition were associated with differences in scaffold compressive behavior, with CH–G–HA180 exhibiting comparatively stable modulus retention across hydrated conditions. Data are presented as mean ± SD (*n* = 3). Statistical significance was evaluated using one-way ANOVA, followed by Tukey’s post hoc test (* *p* < 0.05).

Appropriate mechanical performance is important for maintaining scaffold integrity and influencing scaffold-associated cellular behavior within 3D culture systems [48]. Alterations in ECM stiffness has also been associated with changes in tumor microenvironment organization and cancer progression [48,49]. Accordingly, the compressive modulus of the scaffolds was evaluated under four conditions: dry, wet (DPBS-equilibrated for 6 days), and cell-laden following 6 days of culture with either A549 or PANC-1 cells. This experimental design enabled evaluation of HA molecular weight-associated mechanical behavior under dehydrated, hydrated, and cell-associated culture conditions (Figure 4, Table 2).

**Figure 5:**
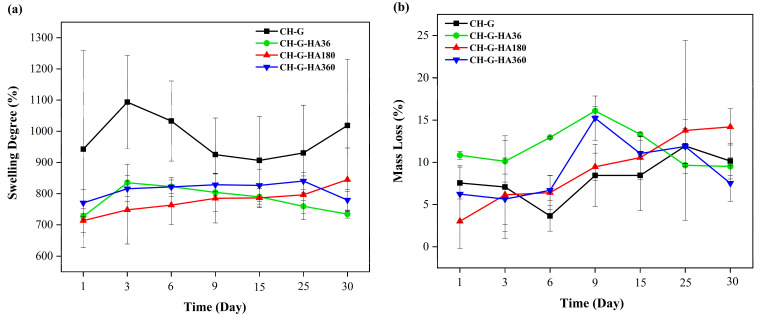
Hydrolytic stability of scaffolds over 30 days. (**a**) Swelling ratio and (**b**) mass loss profiles of CH–G, CH–G–HA36, CH–G–HA180, and CH–G–HA360 in DPBS (pH 7.4) at 37 °C under a 5% CO_2_ atmosphere. The medium MW HA scaffold (CH–G–HA180, red) exhibited comparatively stable swelling and degradation behavior over the incubation period. Data are presented as mean ± SD (*n* = 3).

In the dry state, CH−G−HA36 exhibited the highest compressive modulus (1935 ± 222 kPa), followed by CH−G−HA180 (1009 ± 360 kPa), CH−G−HA360 (551 ± 251 kPa), and the control CH−G (455 ± 21 kPa), as illustrated in Figure 4A. The comparatively high stiffness observed in CH−G−HA36 may be associated with reduced disruption of polymer packing by shorter HA chains, potentially supporting a more organized molecular arrangement within the dehydrated network [27,50,51]. Conversely, the incorporation of HMW-HA (CH−G−HA360) was associated with reduced dry-state stiffness, possibly reflecting increased chain entanglement and less compact polymer packing. Overall, HA-containing scaffolds exhibited higher dry-state stiffness compared with the HA-free control scaffold. Following hydration under DPBS-equilibrated conditions, the stiffness hierarchy changed substantially. CH−G−HA180 exhibited the highest wet-state modulus (298 ± 125 kPa), followed by CH−G−HA360 (206 ± 23 kPa), CH−G−HA36 (133 ± 32 kPa), and CH−G (68 ± 21 kPa), as shown in Figure 4B. The comparatively higher hydrated stiffness observed in CH−G−HA180 corresponded with its relatively homogeneous pore architecture (Figure 3c) and moderate swelling behavior (Figure 5a), suggesting that medium-molecular-weight HA supported balanced hydration-associated network organization [52]. In contrast, CH−G−HA36 exhibited lower hydrated stiffness, whereas CH−G−HA360 demonstrated greater variability in structural organization. Collectively, these observations indicate that HA molecular weight influenced hydration-associated compressive behavior within the scaffold system. Broad contextual literature comparisons of reported mechanical properties for representative cancer-related biomaterial platforms and tumor models are provided in Supplementary Table S1 to situate the current findings within the wider field.

Following 6 days of A549 cell culture, CH−G−HA360 exhibited the highest compressive modulus (292 ± 21 kPa), followed by CH−G−HA180 (250 ± 45 kPa). Conversely, CH−G (186 ± 33 kPa) and CH−G−HA36 (178 ± 55 kPa) demonstrated comparatively lower stiffness values (Figure 4C). The comparatively higher stiffness observed in CH−G−HA360 following A549 culture may reflect differences in scaffold-associated cell interactions and localized structural reorganization within the HA-containing matrices [48,53,54]. In contrast, CH−G and CH−G−HA36 exhibited comparatively limited changes in compressive behavior following culture. Despite exhibiting a lower modulus than CH−G−HA360, CH−G−HA180 consistently supported the formation of a compact spheroid-like organization, suggesting that scaffold architecture and pore interconnectivity may contribute importantly to multicellular organization in addition to bulk compressive stiffness.

Cell-associated stiffening was more pronounced in PANC-1–laden scaffolds. CH−G−HA360 (HMW-HA) again exhibited the highest modulus (400 ± 72 kPa), followed by CH−G−HA180 (241 ± 79 kPa), CH−G−HA36 (197 ± 39 kPa), and CH−G (152 ± 54 kPa) (Figure 4D). The increased stiffness observed in HA-containing scaffolds following PANC-1 culture is consistent with scaffold-associated structural changes occurring during prolonged culture [55,56,57]. In particular, the comparatively high modulus observed in CH−G−HA360 suggests that HMW-HA may influence cell-associated mechanical behavior within the scaffold system under these culture conditions. Collectively, these observations indicate that HA molecular weight was associated with differences in compressive behavior following culture with distinct cancer cell types.

Although minor differences in scaffold geometry were present among certain cell-laden groups, compressive modulus values were derived from dimensionally normalized stress–strain relationships, thereby enabling a relative comparison of bulk mechanical behavior across scaffold conditions under identical testing parameters. Nevertheless, potential influences of scaffold geometry on hydration behavior and cell distribution should be considered when interpreting cell-laden mechanical responses.

### 3.7. Hydration Stability and Culture-Associated Changes in Compressive Behavior

Hydration stability and relative changes in the scaffold’s compressive behavior were further evaluated (Table 3). Hydration retention varied across scaffold formulations, reflecting variations in scaffold organization associated with HA molecular weight. Fold-change analysis of the compressive modulus revealed formulation-dependent differences in culture-associated mechanical behavior. Notably, the CH–G scaffold showed the largest relative increase in stiffness during culture, whereas the CH–G–HA180 scaffold demonstrated comparatively minimal changes in compressive modulus over the same period.

### 3.8. Swelling and Degradation Behavior

The swelling and degradation behavior of scaffolds influences their structural integrity and biological functionality during prolonged culture. Swelling facilitates fluid absorption, nutrient diffusion, and waste elimination, whereas controlled degradation provides sustained mechanical support and allows ECM deposition [43]. Because HA is highly hydrophilic and susceptible to hydrolytic and enzymatic degradation [52,53], we anticipated that its HA MW would influence scaffold hydration and degradation dynamics. Accordingly, all scaffold variants were evaluated for swelling behavior and mass loss over 30 days in DPBS (pH 7.4) to assess hydration-associated structural stability under physiologically relevant aqueous conditions.

Swelling behavior exhibited apparent variations with HA MW (Figure 5a). The chitosan–gelatin control (CH−G) exhibited the highest initial swelling (~1000%), followed by an irregular decline, suggesting less regulated hydration behavior in the absence of HA [58,59]. CH−G−HA36 displayed increased swelling until day 3, followed by a gradual decrease, indicating that lower-molecular-weight HA promoted early hydration but was associated with comparatively reduced structural stability over time. Conversely, CH−G−HA180 exhibited a gradual, sustained increase in swelling over 30 days, suggesting comparatively stable hydration behavior within the scaffold network. CH−G−HA360 exhibited high swelling, with an overall increasing trend until day 25, followed by a slight decline, potentially reflecting localized relaxation or partial degradation. Overall, these observations indicate that HA molecular weight influenced hydration-associated scaffold behavior, with CH−G−HA180 exhibiting the most stable swelling profile over prolonged incubation [52,58,59].

Degradation trends generally paralleled the swelling behaviors (Figure 5b). CH−G−HA36 exhibited the greatest mass loss over time, consistent with its comparatively irregular and highly hydrated structure. In contrast, CH−G−HA180 demonstrated the greatest structural stability over 30 days, supporting the interpretation that the tested intermediate HA molecular-weight condition contributed to comparatively stable scaffold organization [60]. CH−G−HA360 exhibited a non-monotonic degradation profile, characterized by increased mass loss between days 6 and 9, followed by relative stabilization, suggesting heterogeneous degradation behavior within the scaffold network. The control sample (CH−G) also exhibited irregular degradation behavior, consistent with its comparatively less organized architecture. Collectively, these findings suggest that HA molecular weight influenced degradation-associated behavior by modulating scaffold hydration and physical network organization [61,62].

Importantly, all scaffolds underwent identical glutaraldehyde crosslinking and glycine neutralization conditions; therefore, the comparatively enhanced stability of CH−G−HA180 is more likely associated with differences in physical network organization rather than with variations in covalent crosslinking density. Medium-MW HA may promote a comparatively balanced chain entanglement and non-covalent interactions, such as hydrogen bonding and ionic PECs, thereby contributing to sustained structural stability during prolonged hydration. Similar gradual degradation behavior has previously been reported in chitosan–gelatin scaffold systems [14]. Although degradation in the present study was evaluated under DPBS conditions and therefore primarily reflects hydrolytic and physical dissolution processes, biological environments may additionally introduce enzymatic degradation pathways, such as hyaluronidase-mediated HA cleavage, which could further influence scaffold degradation kinetics [63]. Overall, the comparatively stable swelling and degradation behavior observed in CH−G−HA180 supported its suitability for prolonged scaffold-associated multicellular culture applications.

### 3.9. Metabolic Activity (CCK-8 Assay)

Relative metabolic activity of A549 and PANC-1 cells cultured on scaffolds with various HA molecular weights was evaluated on days 1, 3, and 6 using the CCK-8 assay. Data were normalized to the corresponding intra-group Day 1 baseline for each experimental condition to evaluate temporal changes in metabolic activity within the 3D scaffold environment.

**Figure 6:**
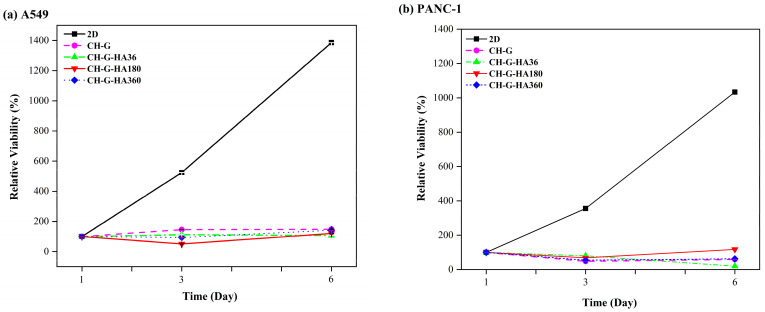
Relative metabolic activity of (**a**) A549 and (**b**) PANC-1 cells cultured on scaffolds for 1, 3, and 6 days as determined using the CCK-8 assay. Values were normalized to the corresponding intra-group Day 1 baseline for each experimental condition. Data points represent mean values, and complete data sets (mean ± SD) are provided in Supplementary Materials Table S2.

A549 cells exhibited measurable metabolic activity across all scaffold formulations, with scaffold-dependent temporal trends observed over the culture period (Figure 6a). The HA-free scaffold (CH−G) demonstrated a progressive increase in relative metabolic activity, reaching 148% by day 6. CH−G−HA360 exhibited a comparable trend, reaching 141% by day 6. CH−G−HA36 demonstrated a moderate increase in metabolic activity (112% on day 3) and experienced a slight reduction by day 6. Conversely, CH−G−HA180 exhibited a transient reduction in relative metabolic activity to 51% at day 3, followed by a significant recovery to 120% by day 6. Overall, scaffolds incorporating medium- and high-MW HA (CH−G−HA180 and CH−G−HA360) maintained comparatively higher metabolic activity in A549 cells over the culture period. These observations suggest that scaffold architecture, hydration behavior, and structural stability may influence the temporal metabolic response of A549 cells within the scaffold environment [52].

PANC-1 cells exhibited comparatively greater sensitivity to scaffold formulation (Figure 6b). Among the scaffold groups, CH−G−HA180 demonstrated the greatest increase in relative metabolic activity over time, increasing from 68% on day 3 to 117% on day 6. CH−G exhibited comparatively lower metabolic activity at 59% by day 6, whereas CH−G−HA36 decreased substantially from 100% to 19% between days 1 and 6. CH−G−HA360 demonstrated moderate metabolic activity over the culture period, reaching 62% by day 6. Collectively, these findings indicate that differences in scaffold hydration behavior, degradation characteristics, and physical organization were associated with distinct metabolic activity profiles between A549 and PANC-1 cells cultured within the scaffold system [64,65].

### 3.10. Cell Morphology and Scaffold Interaction (SEM Imaging)

**Figure 7:**
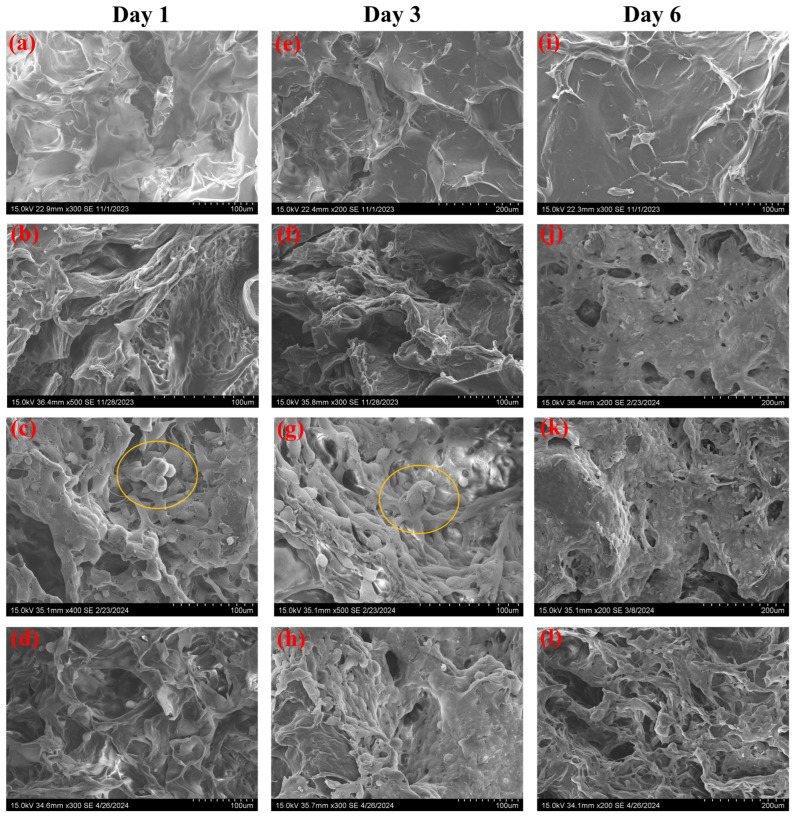
Morphological progression of A549 cells on scaffolds over time. SEM micrographs display scaffold-associated cellular organization on CH–G (**a**,**e**,**i**), CH–G–HA36 (**b**,**f**,**j**), CH–G–HA180 (**c**,**g**,**k**), and CH–G–HA360 (**d**,**h**,**l**) after 1, 3, and 6 days of culture. Magnifications vary across panels (scale bars: 100–200 μm) to highlight structural architectures and cellular features. Distinct cell-scaffold interactions and early cellular aggregation are evident on CH–G–HA180 (**c**,**g**) relative to the other formulations. Yellow circles highlight representative cellular aggregates observed on CH–G–HA180.

**Figure 8:**
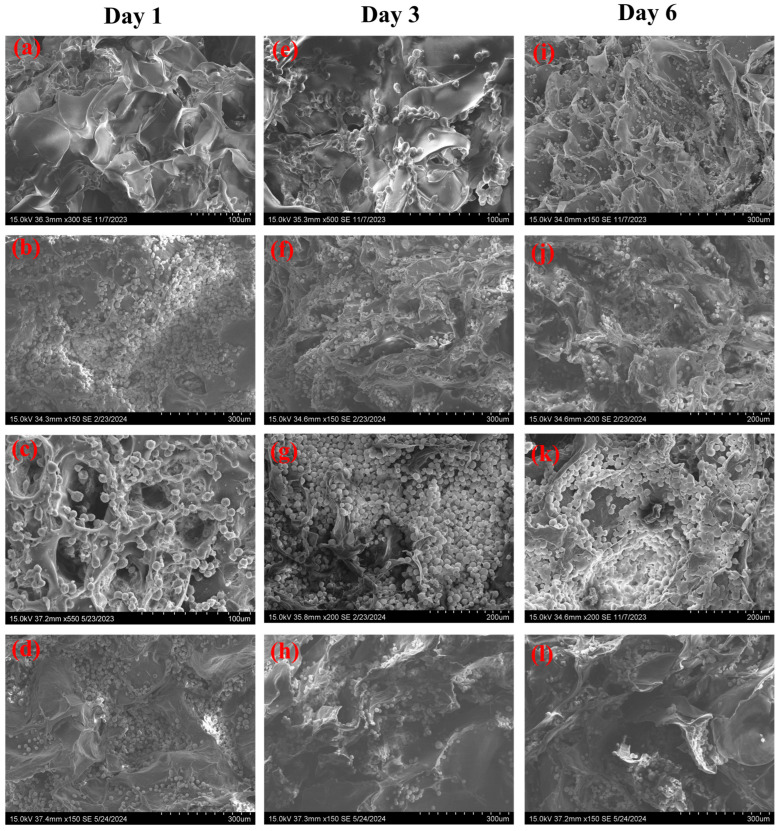
Morphological progression of PANC-1 cells on scaffolds over time. SEM micrographs display scaffold-associated cellular organization on CH–G (**a**,**e**,**i**), CH–G–HA36 (**b**,**f**,**j**), CH–G–HA180 (**c**,**g**,**k**), and CH–G–HA360 (**d**,**h**,**l**) after 1, 3, and 6 days of culture. Magnifications vary across panels (scale bars: 100–300 μm) to capture architectural features and cellular distribution. More evident scaffold-associated clustering and cellular extension into the porous network are observed on CH–G–HA180.

**Figure 9:**
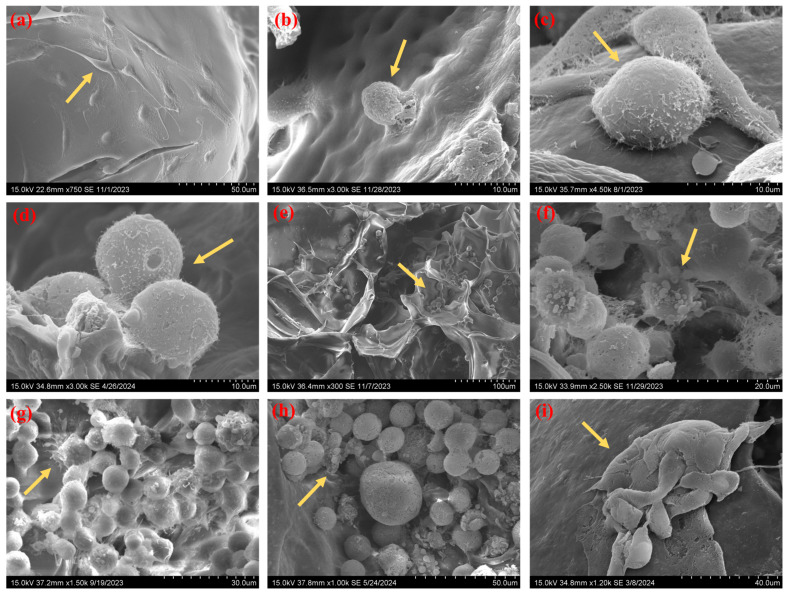
Early attachment and scaffold-associated organization of cancer cells on the fabricated matrices. SEM micrographs display (**a**–**d**) A549 and (**e**–**h**) PANC-1 cell morphology on CH–G, CH–G–HA36, CH–G–HA180, and CH–G–HA360 scaffolds after 24 h of culture. Yellow arrows indicate representative cells and cellular extensions. By day 6, a compact, spheroid-like multicellular organization is visible in A549 cells on CH–G–HA180 (**i**). Scale bars are adjusted to resolve specific cellular features.

To observe cell attachment, spreading, and scaffold-associated organization, SEM imaging was conducted on A549 and PANC-1 cells cultured within scaffolds on days 1, 3, and 6 (Figure 7, Figure 8 and Figure 9). The SEM observations showed cell-type- and formulation-dependent differences in morphology, surface coverage, and scaffold-associated cellular organization. A549 cells cultured on the HA-free scaffold (CH–G) showed comparatively sparse surface and elongated morphologies through day 6 (Figure 7a,e,i). Although metabolic activity was detected by CCK-8 analysis, SEM observations suggested limited scaffold-associated cellular organization in the absence of HA.

CH−G−HA36 showed marked temporal changes in A549 cell morphology. The LMW-HA scaffold displayed sparse cellular distribution on days 1 and 3 (Figure 7b,f), followed by increased surface coverage by day 6 (Figure 7j). This trend may be associated with the less stable hydration and degradation behavior observed for CH−G−HA36. In comparison to CH−G and CH−G−HA36, scaffolds containing medium- and high-MW HA (CH−G−HA180 and CH−G−HA360) supported more evident cell attachment and cell–cell association. A549 cells exhibited rounded, well-anchored morphologies, with visible cellular extensions observed at higher magnification (Figure 9c,d). These features were less evident in CH−G and only modestly observed in CH−G−HA36, suggesting that HA-containing scaffolds, particularly CH−G−HA180 and CH−G−HA360, provided more favorable microstructural environments for scaffold-associated cellular organization.

CH−G−HA180 showed early cellular aggregation by day 1, denser multicellular assemblies by day 3 (Figure 7c,g,k), and compact spheroid-like organization by day 6 (Figure 9i). Representative SEM-based morphometric analysis of A549 spheroid-like structures at day 6 further supported this observation, showing measurable spheroid-like features including area, Feret diameter, circularity, aspect ratio, and solidity (Supplementary Table S3). These measurements are presented as supportive morphological descriptors rather than definitive evidence of tumor phenotype. CH−G−HA360 exhibited apparent initial attachment and cellular extensions; however, cells formed smaller and more spatially confined aggregates rather than larger spheroid-like structures (Figure 7d,h,l). Together, these SEM observations suggest that scaffold architecture and HA molecular weight were associated with distinct patterns of A549 scaffold-associated organization.

PANC-1 cells cultured on HA-free scaffolds exhibited poor attachment and minimal surface interaction. Small grape-like aggregates were observed by day 3 (Figure 8e), but were less evident by day 6 (Figure 8i), suggesting reduced scaffold-associated organization in the absence of HA. Cells on CH−G−HA36 displayed some protrusive features; however, they remained sparsely distributed and poorly organized (Figure 8b,f,j), which may be associated with the LMW-HA scaffold’s less stable swelling and degradation behavior. In contrast to CH−G, HA-containing scaffolds, particularly CH−G−HA180 and CH−G−HA360, supported more evident cell attachment, protrusive morphology, and scaffold-associated clustering. On CH−G−HA180 and CH−G−HA360 scaffolds, PANC-1 cells formed interconnected cellular clusters with visible filopodia- and lamellipodia-like extensions (Figure 9g,h). These features were less pronounced on CH−G−HA36 and largely absent on CH−G, suggesting that HA molecular weight influenced scaffold-associated PANC-1 morphology and organization [57].

CH−G−HA180 supported the most evident cellular distribution within the porous architecture, with interconnected clusters extending into the scaffold pores (Figure 8c,g,k). Individual cells displayed membrane extensions (Figure 9g), consistent with active engagement of the scaffold. Cells on CH−G−HA360 organized into larger, more compact aggregates with irregular, protrusion-rich surfaces (Figure 8d,h,l). Although attachment and protrusive morphology were evident, the denser scaffold architecture of CH−G−HA360 appeared to be associated with more spatially confined cellular organization (Figure 9h) compared with the comparatively more distributed organization observed in CH−G−HA180.

Collectively, SEM observations indicated that HA molecular weight was associated with distinct scaffold-associated organization patterns in both cancer cell lines. Scaffolds lacking HA (CH−G) or incorporating low-MW HA (CH−G−HA36) showed comparatively limited or less organized cell distribution, whereas scaffolds containing medium- and high-MW HA (CH−G−HA180 and CH−G−HA360) supported more evident attachment, protrusive morphology, and multicellular organization. Overall, these observations suggest that CH−G−HA180 provided a balanced scaffold microarchitecture for supporting scaffold-associated multicellular organization under the tested culture conditions. A broad comparison of the contextual literature on representative 3D tumor spheroid and scaffold-based culture systems, including HA-containing and related biomaterial platforms, is provided in Supplementary Table S4 to contextualize the present work within the broader development of biomaterial-assisted 3D tumor culture systems.

## 4. Discussion

The present study demonstrates that the molecular weight of hyaluronic acid (HA MW) influences both the physical properties and the scaffold-associated cellular organization of chitosan–gelatin–HA matrices under constant formulation and crosslinking conditions. By systematically varying HA chain length while maintaining an identical overall scaffold composition, differences were observed in pore architecture, hydration behavior, degradation stability, compressive properties, and spatial cellular organization. Among the investigated formulations, medium-MW HA (CH–G–HA180) consistently exhibited balanced structural and functional characteristics across multiple characterization conditions. Collectively, these findings suggest that HA molecular weight is an important structural parameter that influences matrix behavior and associated cellular responses in this 3D biomaterial system.

### 4.1. Influence of HA Molecular Weight on Scaffold Architecture and Physical Behavior

Architectural characterization revealed that HA molecular weight influences pore formation in the chitosan–gelatin matrix. The HA-free control (CH–G) produced irregular pore configurations, which may reflect the baseline packing behavior of the unmodified polymer backbone. Conversely, the incorporation of medium-MW HA (CH–G–HA180) exhibited in a more homogeneous microarchitecture characterized by uniform pore dimensions, porosity, and interconnectivity, associated with favorable pore organization and localized spatial cellular distribution [44,66,67]. Low-MW HA (CH–G–HA36) resulted in heterogeneous and structurally variable matrices, likely due to higher macromolecular chain mobility, whereas high-MW HA (CH–G–HA360) induced localized densification, which may be attributed to extensive physical chain entanglement. These architectural trends demonstrate that variation in HA chain length under constant compositional and crosslinking conditions alters the physical landscape of the 3D matrix, thereby providing a distinct design variable relative to traditional concentration-dependent studies [68].

This physical reinforcement further modulated the matrices’ fluid transport and hydrolytic properties. CH–G–HA36 (LMW-HA) demonstrated rapid swelling kinetics and accelerated mass loss, potentially driven by greater water ingress and reduced physical chain entanglement, which may contribute to reduced network stability. In contrast, CH–G–HA180 (MMW-HA) exhibited stable hydration capacity and minimal degradation, suggesting balanced physical packing and network organization. CH–G–HA360 (HMW-HA) restricted early swelling but displayed intermittent mass loss, which correlated with its localized densification. Importantly, because identical glutaraldehyde crosslinking conditions were used across all groups, these differences arise from physical and spatial variations introduced by HA chain length rather than from chemical modifications, in contrast to alternative platforms that achieve stability through additive chemical crosslinking [14].

### 4.2. Scaffold-Associated Temporal Metabolic Activity and Cellular Organization

Mechanical and morphological characterization across dry, hydrated, and cell-laden states further indicated that matrix stiffness and cellular spatial organization varied with HA MW. Although the chemical crosslink density was maintained constant, the compressive moduli altered with HA chain length, attributable to differences in polymer packing (dry state), resistance to hydration (hydrated state), and cell-associated structural changes (cell-laden state). High-MW HA (CH–G–HA360) supported substantial stiffening during culture, which correlated with the comparatively denser scaffold architecture. CH–G–HA180, in contrast, maintained relatively stable and moderate stiffness across all examined conditions, indicating a balance between hydration behavior and physical entanglement.

These distinct structural environments correlated with cell-type–specific spatial organization and temporal metabolic responses [54]. A549 cells maintained metabolic activity across all scaffold variants but formed more compact, localized aggregates on CH–G–HA180. Conversely, PANC-1 cells exhibited a more selective spatial distribution, forming interconnected multicellular clusters primarily within the medium-MW system. The scaffold stiffness range (70–400 kPa) is higher than that of traditional soft hydrogels (0.5–10 kPa) and the baseline values reported for soft-tissue bulk tumor ECM (0.5–40 kPa) [69,70]. This elevated mechanical profile may therefore be relevant for investigating comparatively rigid matrix environments, while also emphasizing that variations in testing methodology must be considered when comparing bulk mechanical values across different 3D platforms.

### 4.3. Proposed Material–Structure Relationship Within the Scaffold System

The collective findings suggest that HA’s macromolecular size influences the physical presentation of architectural and mechanical cues within the scaffold system. Hydration retention and fold-change stiffness analysis provide additional insight into these physical interactions. While bulk modulus values describe the baseline matrix stiffness, the fold-change values reflect the magnitude of stiffness alterations during culture relative to the initial hydrated state. The HA-free matrix (CH–G) exhibited the largest relative fold change in stiffness, suggesting localized physical restructuring or compaction within a less-hydrated polymer network. In contrast, CH–G–HA180 (MMW-HA) exhibited minimal mechanical fold-change, suggesting that its comparatively uniform pore distribution and balanced polymer entanglement may provide a more stable structural environment that requires less physical rearrangement during culture.

Because the total polymer composition and crosslinking conditions were kept identical across all experimental groups, these findings suggest that HA chain length functions as an effective structural tuning parameter within the scaffold system. The observed variations in pore stability, swelling behavior, and compressive properties suggest that modulating HA molecular weight may offer an accessible strategy for adjusting the matrix’s physical behavior without introducing alternative crosslinking chemistries or compositional modifications.

### 4.4. Study Limitations and Future Perspectives

While this study identified clear correlations between HA molecular weight and scaffold physical behavior, several limitations should be acknowledged. First, although differences in cell distribution, protrusion morphology, and multicellular organization were observed across the HA-containing scaffold groups, receptor-level signaling pathways, such as CD44- or RHAMM-associated interactions, were not empirically investigated in this work despite their documented relevance in the literature [31,71,72]. Accordingly, the present system should be interpreted primarily as a structurally tunable physical platform rather than a validated molecular signaling model. Second, temporal metabolic activity was evaluated using the CCK-8 assay with liquid-only blank subtraction. Because scaffold-specific background absorbance was not independently evaluated at each time point, the metabolic activity data should be interpreted as relative behavioral trends rather than as an absolute quantification of cell number. Furthermore, formal assessments of normality and homogeneity of variance assumptions were not performed prior to ANOVA, given the limited sample size used in several characterization experiments (*n* = 3). Consequently, the statistical analyses should be interpreted as exploratory comparisons intended to support observed experimental trends rather than as definitive population-level inferences. Degradation analysis was performed strictly under hydrolytic DPBS conditions and therefore does not fully represent the enzymatic degradation processes that may occur in dynamic biological environments.

Future studies incorporating scaffold-specific blank controls, complementary cell viability assays, enzymatic degradation models, and molecular-level analyses may further clarify the mechanistic basis underlying the observed scaffold-associated cellular responses. Within these stated boundaries, the present scaffold system provides a physically defined baseline platform for investigating HA-molecular-weight-dependent variations in scaffold architecture and associated cellular organization in 3D culture environments.

## 5. Conclusions

This study demonstrates that hyaluronic acid (HA) influences the physicochemical and mechanical behavior of 3D chitosan–gelatin–HA matrices under constant chemical composition and crosslinking conditions. Systematic variation in HA chain length was associated with differences in pore architecture, swelling behavior, hydrolytic degradation stability, and compressive properties under dry, hydrated, and cell-laden conditions. Among the investigated formulations, medium-MW HA (CH–G–HA180) consistently exhibited comparatively uniform pore organization, stable hydration behavior, and moderate mechanical stability across multiple characterization conditions.

Furthermore, these distinct scaffold architectures were associated with differences in scaffold-associated cellular organization and temporal trends in metabolic activity for A549 and PANC-1 cells in 3D culture. The comparatively elevated stiffness range observed in the present scaffold system may also be relevant for investigating rigid or densified matrix environments compared with conventional soft hydrogel systems. Because these variations were achieved without altering the overall scaffold composition or introducing alternative crosslinking chemistries, the findings suggest that HA molecular weight serves as an accessible structural tuning parameter in this biomaterial platform. Within the limitations of the present study, this system provides a reproducible, physically defined 3D scaffold model to investigate HA molecular-weight-dependent differences in scaffold organization and associated scaffold-related cellular responses.

## Figures and Tables

**Table 1:** Compositional parameters and nomenclature for the chitosan–gelatin base scaffold (control) and the isocompositional HA-containing scaffold series with varying HA molecular weights ^†^.

Scaffold Code	Description	HA MW (kDa)	CH Conc. (% *w*/*v*)	G Conc. (% *w*/*v*)	HA Conc. (% *w*/*v*)	Total Conc. (% *w*/*v*)
CH–G	Chitosan–Gelatin (Control)	None	2	1	N/A	3
CH–G–HA36	Chitosan–Gelatin–HA36	36 (LMW)	2	1	1	4
CH–G–HA180	Chitosan–Gelatin–HA180	180 (MMW)	2	1	1	4
CH–G–HA360	Chitosan–Gelatin–HA360	360 (HMW)	2	1	1	4

^†^ All scaffolds were stabilized by crosslinking with 1% (*v*/*v*) glutaraldehyde solution in Milli-Q water for 4–5 h. Abbreviations: CH, chitosan; G, gelatin; HA, hyaluronic acid; MW, molecular weight; LMW, low molecular weight; MMW, medium molecular weight; HMW, high molecular weight; % *w*/*v*, weight/volume percentage; N/A, not applicable.

**Table 2:** Morphological, architectural, and compressive properties of the fabricated scaffolds. Mean pore size, porosity, and compressive modulus under dry, wet (DPBS-equilibrated), and cell-laden conditions are presented as mean ± standard deviation (SD). Replicate numbers were *n* = 3 for pore size and compressive modulus, and *n* = 12 for porosity.

Scaffold	Pore Size (µm)	Porosity (%)	Compressive Modulus (kPa)			
			Dry	Wet	A549 Cells	PANC-1 Cells
CH–G	227 ± 50	60	455 ± 21	68 ± 21	186 ± 33	152 ± 54
CH–G–HA36	281 ± 60	63	1935 ± 222	133 ± 32	178 ± 55	197 ± 39
CH–G–HA180	100 ± 20	83	1009 ± 360	298 ± 125	250 ± 45	241 ± 79
CH-G-HA360	216 ± 50	66	551 ± 251	206 ± 23	292 ± 21	400 ± 72

**Table 3:** Hydration retention and relative changes in compressive behavior of the scaffold formulations during culture.

Formulation	Hydration Retention (%)	A549 Stiffening (Fold-Change)	PANC-1 Stiffening (Fold-Change)
	(*Modulus _Wet_*/*Modulus _Dry_*) × 100	(*Modulus _Cell-Laden_*/*Modulus _Wet_*)	(*Modulus _Cell-Laden_*/*Modulus _Wet_*)
CH−G (Control)	15%	2.73-fold	2.23-fold
CH−G−HA36 (LMW-HA)	7%	1.33-fold	1.48-fold
CH−G−HA180 (MMW-HA)	29%	0.83-fold	0.80-fold
CH−G−HA360 (HMW-HA)	37%	1.41-fold	1.94-fold

Note: Fold-change values represent the ratio of the compressive modulus of cell-laden scaffolds relative to the corresponding acellular hydrated scaffold modulus after 6 days in culture.

## Data Availability

The original contributions presented in this study are included in the article/Supplementary Materials. Further inquiries can be directed to the corresponding authors.

## Supplementary Materials

The following supporting information can be downloaded at: https://www.mdpi.com/article/10.3390/polym18141703/s1, Table S1. Broad contextual literature comparison of reported mechanical properties for representative cancer-related biomaterial platforms and tumor-model systems employing different materials, testing methodologies, and experimental conditions. Values are presented for contextual reference only and should not be interpreted as directly comparable across studies; Table S2. Relative metabolic activity of A549 and PANC-1 cells cultured on scaffolds over time, as determined by the CCK-8 assay. Data are expressed as a percentage relative to the corresponding intra-group Day 1 baseline value (mean ± SD; *n* = 5), representing the relative fold-change in metabolic activity over the 6-day culture period; Table S3. Representative SEM-based morphometric analysis of A549 spheroid-like structures observed on CH–G–HA180 scaffolds at day 6; Table S4. Broad contextual overview of representative three-dimensional tumor spheroid and scaffold-based culture systems incorporating hyaluronic acid (HA) or related biomaterial components (2012–present). References [15,55,68,73,74,75,76,77,78,79,80] are cited in Tables S1 and S4 in the Supplementary Materials; Figure S1. Physical appearance (macroscale) of the polymer solutions; Figure S2. Macroscale morphology of freeze-dried scaffolds: (a) CH–G, (b) CH–G–HA36 (LMW-HA), (c) CH–G–HA180 (MMW-HA), and (d) CH–G–HA360 (HMW-HA); Figures S3–S6: Stress–strain curves for CH–G, CH–G–HA36, CH–G–HA180, and CH–G–HA360 scaffolds under dry, wet (DPBS-equilibrated), and cell-laden (A549 and PANC-1 cells) experimental conditions, from which the slope in the linear region was determined to be the compressive modulus.

## References

[1] Pickup M.W., Mouw J.K., Weaver V.M. (2014). The extracellular matrix modulates the hallmarks of cancer. EMBO Rep..

[2] Anderson N.M., Simon M.C. (2020). The tumor microenvironment. Curr. Biol..

[3] Ashworth J.C., Cox T.R. (2024). The importance of 3D fibre architecture in cancer and implications for biomaterial model design. Nat. Rev. Cancer.

[4] Li Y., Khuu N., Prince E., Tao H., Zhang N., Chen Z., Gevorkian A., McGuigan A.P., Kumacheva E. (2020). Matrix stiffness-regulated growth of breast tumor spheroids and their response to chemotherapy. Biomacromolecules.

[5] Doyle A.D., Wang F.W., Matsumoto K., Yamada K.M. (2009). One-dimensional topography underlies three-dimensional fibrillar cell migration. J. Cell Biol..

[6] Pathak A., Kumar S. (2012). Independent regulation of tumor cell migration by matrix stiffness and confinement. Proc. Natl. Acad. Sci. USA.

[7] Bhattacharya D.S., Svechkarev D., Souchek J., Hill T.K., Taylor M., Natarajan A., Mohs A.M. (2017). Impact of structurally modifying hyaluronic acid on CD44 interaction. J. Mater. Chem. B.

[8] Kwon M.Y., Wang C., Galarraga J.H., Puré E., Han L., Burdick J.A. (2019). Influence of hyaluronic acid modification on CD44 binding towards the design of hydrogel biomaterials. Biomaterials.

[9] Yu C., Schimelman J., Wang P., Miller K.L., Ma X., You S., Guan J., Sun B., Zhu W., Chen S. (2020). Photopolymerizable biomaterials and light-based 3D printing strategies for biomedical applications. Chem. Rev..

[10] Tang J.D., Caliari S.R., Lampe K.J. (2018). Temperature-dependent complex coacervation of engineered elastin-like polypeptide and hyaluronic acid polyelectrolytes. Biomacromolecules.

[11] Erickson A.E., Lan Levengood S.K., Sun J., Chang F.C., Zhang M. (2018). Fabrication and characterization of chitosan–hyaluronic acid scaffolds with varying stiffness for glioblastoma cell culture. Adv. Healthc. Mater..

[12] Lalevée G., Sudre G., Montembault A., Meadows J., Malaise S., Crépet A., David L., Delair T. (2016). Polyelectrolyte complexes via desalting mixtures of hyaluronic acid and chitosan—Physicochemical study and structural analysis. Carbohydr. Polym..

[13] Liu H., Mao J., Yao K., Yang G., Cui L., Cao Y. (2004). A study on a chitosan-gelatin-hyaluronic acid scaffold as artificial skin in vitro and its tissue engineering applications. J. Biomater. Sci. Polym. Ed..

[14] Re F., Sartore L., Moulisova V., Cantini M., Almici C., Bianchetti A., Chinello C., Dey K., Agnelli S., Manferdini C. (2019). 3D gelatin-chitosan hybrid hydrogels combined with human platelet lysate highly support human mesenchymal stem cell proliferation and osteogenic differentiation. J. Tissue Eng..

[15] Serafín A., Culebras M., Collins M.N. (2023). Synthesis and evaluation of alginate, gelatin, and hyaluronic acid hybrid hydrogels for tissue engineering applications. Int. J. Biol. Macromol..

[16] Mugnaini G., Gelli R., Mori L., Bonini M. (2023). How to cross-link gelatin: The effect of glutaraldehyde and glyceraldehyde on the hydrogel properties. ACS Appl. Polym. Mater..

[17] Kirdponpattara S., Khamkeaw A., Sanchavanakit N., Pavasant P., Phisalaphong M. (2015). Structural modification and characterization of bacterial cellulose–alginate composite scaffolds for tissue engineering. Carbohydr. Polym..

[18] (2016). Standard Test Method for Compressive Properties of Rigid Cellular Plastics.

[19] Doustdar F., Olad A., Ghorbani M. (2022). Effect of glutaraldehyde and calcium chloride as different crosslinking agents on the characteristics of chitosan/cellulose nanocrystals scaffold. Int. J. Biol. Macromol..

[20] Kim I.-Y., Seo S.-J., Moon H.-S., Yoo M.-K., Park I.-Y., Kim B.-C., Cho C.-S. (2008). Chitosan and its derivatives for tissue engineering applications. Biotechnol. Adv..

[21] Attasgah R.B., Velasco-Rodríguez B., Pardo A., Fernández-Vega J., Arellano-Galindo L., Rosales-Rivera L.C., Prieto G., Barbosa S., Soltero J.F.A., Mahmoudi M. (2022). Development of functional hybrid scaffolds for wound healing applications. iScience.

[22] Qian Y.-F., Zhang K.-H., Chen F., Ke Q.-F., Mo X.-M. (2011). Cross-linking of gelatin and chitosan complex nanofibers for tissue-engineering scaffolds. J. Biomater. Sci. Polym. Ed..

[23] Li Z., Li Q., Cao W., Zhan J., He Y., Xing X., Ding C., Wang L., Qiu X. (2024). A Strongly Robust Chitosan-Based Programmed Control Functional Hydrogel Improved Mitochondrial Function and Pro-Vascularization for Adaptive Repair of Myocardial Infarction. Adv. Funct. Mater..

[24] An B., Cui H., Wang M., Li Z., Li J. (2025). Hydrogel tissue adhesive: Adhesion strategy and application. Colloids Surf. B.

[25] Lipari S., Marfoglia A., Sorrentino G., Cazalbou S., Pilloux L., Sacco P., Donati I. (2025). Thermally cured gelatin-methacryloyl hydrogels form mechanically modulating platforms for cell studies. Biomacromolecules.

[26] Tavakoli S., Krishnan N., Mokhtari H., Oommen O.P., Varghese O.P. (2024). Fine-tuning dynamic cross–linking for enhanced 3D bioprinting of hyaluronic acid hydrogels. Adv. Funct. Mater..

[27] Bigi A., Cojazzi G., Panzavolta S., Rubini K., Roveri N. (2001). Mechanical and thermal properties of gelatin films at different degrees of glutaraldehyde crosslinking. Biomaterials.

[28] Yang P., Boer G., Snow F., Williamson A., Cheeseman S., Samarasinghe R.M., Rifai A., Priyam A., Elnathan R., Guijt R. (2025). Test and tune: Evaluating, adjusting and optimising the stiffness of hydrogels to influence cell fate. Chem. Eng. J..

[29] Dicker K.T., Gurski L.A., Pradhan-Bhatt S., Witt R.L., Farach-Carson M.C., Jia X. (2014). Hyaluronan: A simple polysaccharide with diverse biological functions. Acta Biomater..

[30] Borke T., Winnik F.M., Tenhu H., Hietala S. (2015). Optimized triazine-mediated amidation for efficient and controlled functionalization of hyaluronic acid. Carbohydr. Polym..

[31] Hu Z., Ma R., Li D., Wang T., Elmalki M., Li C., Xue Z., Nie G. (2025). Hierarchically constructed a noncovalent chitosan/hyaluronic acid hydrogel with entanglement-enhanced toughness. Int. J. Biol. Macromol..

[32] Thein-Han W., Saikhun J., Pholpramoo C., Misra R., Kitiyanant Y. (2009). Chitosan–gelatin scaffolds for tissue engineering: Physico-chemical properties and biological response of buffalo embryonic stem cells and transfectant of GFP–buffalo embryonic stem cells. Acta Biomater..

[33] Arısoy S., Bux K., Herwig R., Salva E. (2024). Development, Evaluation, and Molecular Dynamics Study of Ampicillin-Loaded Chitosan–Hyaluronic Acid Films as a Drug Delivery System. ACS Omega.

[34] Farris S., Song J., Huang Q. (2010). Alternative reaction mechanism for the cross-linking of gelatin with glutaraldehyde. J. Agric. Food Chem..

[35] Nguyen T.K.L., Nguyen N.D., Dang V.P., Phan D.T., Tran T.H., Nguyen Q.H. (2019). Synthesis of Platinum Nanoparticles by Gamma Co-60 Ray Irradiation Method Using Chitosan as Stabilizer. Adv. Mater. Sci. Eng..

[36] Servaty R., Schiller J., Binder H., Arnold K. (2001). Hydration of polymeric components of cartilage—An infrared spectroscopic study on hyaluronic acid and chondroitin sulfate. Int. J. Biol. Macromol..

[37] Pawariya V., De S., Dutta J. (2024). Synthesis and characterization of citric acid-modified chitosan Schiff base with enhanced antibacterial properties for the elimination of Bismarck Brown R and Rhodamine B dyes from wastewater. Int. J. Biol. Macromol..

[38] Hay M.B., Myneni S.C. (2007). Structural environments of carboxyl groups in natural organic molecules from terrestrial systems. Part 1: Infrared spectroscopy. Geochim. Cosmochim. Acta.

[39] Saito K., Xu T., Ishikita H. (2022). Correlation between C═O stretching vibrational frequency and p*K*_a_ shift of carboxylic acids. J. Phys. Chem. B.

[40] Florczyk S.J., Wang K., Jana S., Wood D.L., Sytsma S.K., Sham J.G., Kievit F.M., Zhang M. (2013). Porous chitosan-hyaluronic acid scaffolds as a mimic of glioblastoma microenvironment ECM. Biomaterials.

[41] Hamidi S., Maton M., Hildebrand F., Gaucher V., Bossard C., Cazaux F., Staelens J.N., Blanchemain N., Martel B. (2025). Design and Evaluation of a Crosslinked Chitosan-Based Scaffold Containing Hyaluronic Acid for Articular Cartilage Reconstruction. Molecules.

[42] Gencoglu M.F., Barney L.E., Hall C.L., Brooks E.A., Schwartz A.D., Corbett D.C., Stevens K.R., Peyton S.R. (2018). Comparative study of multicellular tumor spheroid formation methods and implications for drug screening. ACS Biomater. Sci. Eng..

[43] Abuwatfa W.H., Pitt W.G., Husseini G.A. (2024). Scaffold-based 3D cell culture models in cancer research. J. Biomed. Sci..

[44] Karageorgiou V., Kaplan D. (2005). Porosity of 3D biomaterial scaffolds and osteogenesis. Biomaterials.

[45] Mukasheva F., Adilova L., Dyussenbinov A., Yernaimanova B., Abilev M., Akilbekova D. (2024). Optimizing scaffold pore size for tissue engineering: Insights across various tissue types. Front. Bioeng. Biotechnol..

[46] Hollister S.J. (2005). Porous scaffold design for tissue engineering. Nat. Mater..

[47] O’Brien F.J. (2011). Biomaterials & scaffolds for tissue engineering. Mater. Today.

[48] Deng B., Zhao Z., Kong W., Han C., Shen X., Zhou C. (2022). Biological role of matrix stiffness in tumor growth and treatment. J. Transl. Med..

[49] Massey A., Stewart J., Smith C., Parvini C., McCormick M., Do K., Cartagena-Rivera A.X. (2024). Mechanical properties of human tumour tissues and their implications for cancer development. Nat. Rev. Phys..

[50] Lin C.-C., Metters A.T. (2006). Hydrogels in controlled release formulations: Network design and mathematical modeling. Adv. Drug Deliv. Rev..

[51] Lin J., Pan D., Sun Y., Ou C., Wang Y., Cao J. (2019). The modification of gelatin films: Based on various cross-linking mechanism of glutaraldehyde at acidic and alkaline conditions. Food Sci. Nutr..

[52] Burdick J.A., Prestwich G.D. (2011). Hyaluronic acid hydrogels for biomedical applications. Adv. Mater..

[53] Carvalho M.P., Costa E.C., Miguel S.P., Correia I.J. (2016). Tumor spheroid assembly on hyaluronic acid-based structures: A review. Carbohydr. Polym..

[54] Line Q. (2002). Hyaluronan-CD44s Signaling Regulates Matrix. Cancer Res..

[55] Ermis M., Falcone N., De Barros N.R., Mecwan M., Haghniaz R., Choroomi A., Monirizad M., Lee Y., Song J., Cho H.-J. (2023). Tunable hybrid hydrogels with multicellular spheroids for modeling desmoplastic pancreatic cancer. Bioact. Mater..

[56] Pandol S., Edderkaoui M., Gukovsky I., Lugea A., Gukovskaya A. (2009). Desmoplasia of pancreatic ductal adenocarcinoma. Clin. Gastroenterol. Hepatol..

[57] Shichi Y., Sasaki N., Michishita M., Hasegawa F., Matsuda Y., Arai T., Gomi F., Aida J., Takubo K., Toyoda M. (2019). Enhanced morphological and functional differences of pancreatic cancer with epithelial or mesenchymal characteristics in 3D culture. Sci. Rep..

[58] Engel B.J., Constantinou P.E., Sablatura L.K., Doty N.J., Carson D.D., Farach-Carson M.C., Harrington D.A., Zarembinski T.I. (2015). Multi-layered, hyaluronic acid-based hydrogel formulations suitable for automated 3D high throughput drug screening of cancer-stromal cell co-cultures. Adv. Healthc. Mater..

[59] Chen X., Thibeault S.L. (2016). Cell–cell interaction between vocal fold fibroblasts and bone marrow mesenchymal stromal cells in three-dimensional hyaluronan hydrogel. J. Tissue Eng. Regen. Med..

[60] Muzzarelli R.A., Greco F., Busilacchi A., Sollazzo V., Gigante A. (2012). Chitosan, hyaluronan and chondroitin sulfate in tissue engineering for cartilage regeneration: A review. Carbohydr. Polym..

[61] Madl C.M., Katz L.M., Heilshorn S.C. (2018). Tuning bulk hydrogel degradation by simultaneous control of proteolytic cleavage kinetics and hydrogel network architecture. ACS Macro Lett..

[62] An S., Choi S., Min S., Cho S.-W. (2021). Hyaluronic acid-based biomimetic hydrogels for tissue engineering and medical applications. Biotechnol. Bioprocess Eng..

[63] Stern R., Jedrzejas M.J. (2006). Hyaluronidases: Their genomics, structures, and mechanisms of action. Chem. Rev..

[64] Baker B.M., Chen C.S. (2012). Deconstructing the third dimension–how 3D culture microenvironments alter cellular cues. J. Cell Sci..

[65] Baruffaldi D., Palmara G., Pirri C., Frascella F. (2021). 3D cell culture: Recent development in materials with tunable stiffness. ACS Appl. Bio Mater..

[66] Jaipaew J., Wangkulangkul P., Meesane J., Raungrut P., Puttawibul P. (2016). Mimicked cartilage scaffolds of silk fibroin/hyaluronic acid with stem cells for osteoarthritis surgery: Morphological, mechanical, and physical clues. Mater. Sci. Eng. C.

[67] Sionkowska A., Michalska-Sionkowska M., Walczak M. (2020). Preparation and characterization of collagen/hyaluronic acid/chitosan film crosslinked with dialdehyde starch. Int. J. Biol. Macromol..

[68] Demirel G., Cakıl Y.D., Koltuk G., Aktas R.G., Calıskan M. (2024). The use of hyaluronic acid in a 3D biomimetic scaffold supports spheroid formation and the culture of cancer stem cells. Sci. Rep..

[69] Levental K.R., Yu H., Kass L., Lakins J.N., Egeblad M., Erler J.T., Fong S.F.T., Csiszar K., Giaccia A., Weninger W. (2009). Matrix crosslinking forces tumor progression by enhancing integrin signaling. Cell.

[70] Laklai H., Miroshnikova Y.A., Pickup M.W., Collisson E.A., Kim G.E., Barrett A.S., Hill R.C., Lakins J.N., Schlaepfer D.D., Mouw J.K. (2016). Genotype tunes pancreatic ductal adenocarcinoma tissue tension to induce matricellular fibrosis and tumor progression. Nat. Med..

[71] Toole B.P. (2004). Hyaluronan: From extracellular glue to pericellular cue. Nat. Rev. Cancer.

[72] Caliari S.R., Burdick J.A. (2016). A practical guide to hydrogels for cell culture. Nat. Methods.

[73] Pele K.G., Calderón-Villalba A., Amaveda H., Mora M., Zhang-Zhou J., Pérez M.Á., García-Aznar J.M., Alamán-Díez P., García-Gareta E. (2025). Novel hydrogel-based cancer-on-a-chip models for growth of 3D multi-cellular structures and investigation of early angiogenesis in pancreatic ductal adenocarcinoma. Colloids Surf. B.

[74] Lee A., de Almeida M.S., Milinkovic D., Septiadi D., Taladriz-Blanco P., Loussert-Fonta C., Balog S., Bazzoni A., Rothen-Rutishauser B., Petri-Fink A. (2022). Substrate stiffness reduces particle uptake by epithelial cells and macrophages in a size-dependent manner through mechanoregulation. Nanoscale.

[75] Williams A.H., Hebert A.M., Boehm R.C., Huddleston M.E., Jenkins M.R., Velev O.D., Nelson M.T. (2021). Bioscaffold stiffness mediates aerosolized nanoparticle uptake in lung epithelial cells. ACS Appl. Mater. Interfaces.

[76] Nguyen A.V., Nyberg K.D., Scott M.B., Welsh A.M., Nguyen A.H., Wu N., Hohlbauch S.V., Geisse N.A., Gibb E.A., Robertson A.G. (2016). Stiffness of pancreatic cancer cells is associated with increased invasive potential. Integr. Biol..

[77] Lai J.-Y., Tu I.-H. (2012). Adhesion, phenotypic expression, and biosynthetic capacity of corneal keratocytes on surfaces coated with hyaluronic acid of different molecular weights. Acta Biomater..

[78] Ware M.J., Keshishian V., Law J.J., Ho J.C., Favela C.A., Rees P., Smith B., Mohammad S., Hwang R.F., Rajapakshe K. (2016). Generation of an in vitro 3D PDAC stroma rich spheroid model. Biomaterials.

[79] Monteiro M.V., Gaspar V.M., Mendes L., Duarte I.F., Mano J.F. (2021). Stratified 3D microtumors as organotypic testing platforms for screening pancreatic cancer therapies. Small Methods.

[80] Jiang R., Huang J., Sun X., Chu X., Wang F., Zhou J., Fan Q., Pang L. (2022). Construction of in vitro 3-D model for lung cancer-cell metastasis study. BMC Cancer.

