# Supplementary material for: Hyaluronic Acid Molecular Weight Modulates Chitosan–Gelatin Scaffold Properties and Cancer Cell Organization in 3D Culture
Source: Polymers (Basel). 2026 Jul 10;18(14):1703. doi: 10.3390/polym18141703 (PMC13417349; doi:10.3390/polym18141703)
Supplement: Supplementary file 1 [file polymers-18-01703-s001.zip › polymers-4327431-supplementary.pdf]

Supplementary Materials

Hyaluronic Acid Molecular Weight Modulates Chitosan–Gelatin Scaffold Properties and Cancer Cell Organization in 3D Culture

Leimapokpam Romina Chanu [1], Guo-Chung Dong [2,*] and Ping-Shan Lai [1,3,*]

[1] Doctoral Program in Tissue Engineering and Regenerative Medicine, National Chung Hsing University, Taichung City 402204, Taiwan; romina.annauniversity@gmail.com

[2] Institute of Biomedical Engineering and Nanomedicine, National Health Research Institutes, Zhunan, Miaoli County 35053, Taiwan

[3] Department of Chemistry, National Chung Hsing University, Taichung City 402204, Taiwan

* Correspondence: gcdong@nhri.org.tw (G.-C.D.); pslai@email.nchu.edu.tw (P.-S.L.); Tel.: +886-37-246166 (ext. 37135) (G.-C.D.); +886-4-22840411 (ext. 428) (P.-S.L.)

Table S1. Broad contextual literature comparison of reported mechanical properties for representative cancer-related biomaterial platforms and tumor-model systems employing different materials, testing methodologies, and experimental conditions. Values are presented for contextual reference only and should not be interpreted as directly comparable across studies.

Study (Year)	Material/System	Test Condition	Compressive modulus (kPa)	Cell Type	Remarks
**This study[a]**	CH–G–HA (glutaraldehyde-crosslinked)<br>• CH–G (Control)<br>• CH–G–HA36 (36 kDa; LMW-HA)<br>• CH–G–HA180 (180 kDa; MMW-HA)<br>• CH–G–HA360 (360 kDa; HMW-HA)	Dry	CH–G: 455<br>CH–G–HA36: 1935<br>CH–G–HA180: 1009<br>CH–G–HA360: 551	–	Represents the intrinsic mechanical baseline of the scaffold network. Low-MW HA (CH–G–HA36) exhibited comparatively higher dry compressive stability, which may support practical scaffold handling
		Wet (DPBS-equilibrated, Day 6)	CH–G: 68<br>CH–G–HA36: 133<br>CH–G–HA180: 298<br>CH–G–HA360: 206	–	Medium-MW HA (CH–G–HA180) exhibited comparatively stable hydration-associated mechanical behavior under prolonged aqueous conditions
		Cell-laden (Day 6)	CH–G: 186<br>CH–G–HA36: 178<br>CH–G–HA180: 250<br>CH–G–HA360: 292	A549	Demonstrated that HA MW is associated with differences in cell-laden scaffold mechanical behavior and scaffold-associated multicellular organization in A549 and PANC-1 3D culture conditions
		Cell-laden (Day 6)	CH–G: 152<br>CH–G–HA36: 197<br>CH–G–HA180: 241<br>CH–G–HA360: 400	PANC-1	

Table S1 (continued)

Study (Year)	Material/System	Test Condition	Compressive modulus (kPa)	Cell Type	Remarks
K.G. Pele et al. (2025)	Egg White/Gelatin Hydrogel (5% EW + 5% Gelatin)	Hydrated (Material-only)	G' (Elastic Modulus) ~0.5 kPa (Estimated from Fig. 2C rheology data)	PANC-1	Soft EW/Gelatin hydrogel promotes “grape-like” PANC-1 clusters; demonstrates how matrix stiffness and microstructure jointly dictate 3D cancer cell morphology [73]
M. Ermis et al. (2023)	Methacrylated Hyaluronic Acid / Gelatin (HAMA/GelMA) Hydrogel	Hydrated (Material-only), crosslinked (varying durations: 2s, 5s, 10s)	~3 kPa (2s), ~15 kPa (5s), ~36 kPa (10s)	–	Stiffness tuned via UV crosslinking time to model healthy tissue (2s), desmoplastic PDAC (5s), and hyper-desmoplastic PDAC (10s); represents *initial material property* [55]
A. Serafin et al. (2023)	Alginate, Gelatin, and HA Hybrid Hydrogels (various combinations & concentrations)	Hydrated (Material-only)	~135–331 kPa (Alginate), ~501–572 kPa (Gel:HA)	–	Comprehensive screening of biopolymer combinations demonstrates that HA incorporation can reduce hydrogel stiffness [15]
A. Lee et al. (2022)	Polydimethylsiloxane (PDMS) Substrates (2D)	Material-only (Tensile Test)	~2.7 MPa (Stiff), ~130 kPa (Soft)	A549	Demonstrates A549 cell mechanosensitivity on 2D substrates; optimal particle uptake on softer (~130 kPa) surfaces [74]

Table S1 (continued)

Study (Year)	Material/System	Test Condition	Compressive modulus (kPa)	Cell Type	Remarks
A. H. Williams et al. (2021)	Polyurethane Soft Dendritic Colloid (PU SDC) Nonwoven Mat	Material-only (Tensile Test)	~200 kPa (Soft), ~750 kPa (Stiff)	A549	Tunable, porous 3D membrane; demonstrates increased nanoparticle uptake and IL-8 secretion in A549 cells on softer (~200 kPa) substrates [75]
A. V. Nguyen et al. (2016)	N/A (Intrinsic Cell Stiffness)	Adherent Cell (on Matrigel-coated dish)	~2.4 kPa	PANC-1	Measures the intrinsic stiffness of single PANC-1 cells via AFM; shows a positive correlation between cell stiffness and invasive potential in PDAC [76]

Note: [a]This study. Data represent the characterization of CH–G, CH–G–HA36, CH–G–HA180, and CH–G–HA360 under the experimental conditions described in the main manuscript.

Table S2. Relative metabolic activity of A549 and PANC-1 cells cultured on scaffolds over time, as determined by the CCK-8 assay. Data are expressed as a percentage relative to the corresponding intra-group Day 1 baseline value (mean ± SD; *n* = 5), representing the relative fold-change in metabolic activity over the 6-day culture period.

Sample	Cell Line (s)	Day 1 (% of D1 Ctrl)	Day 3 (% of D1 Ctrl)	Day 6 (% of D1 Ctrl)
**2D Control**	A549	100 ± 0.0	524 ± 0.1	1385 ± 0.1
	PANC-1	100 ± 0.0	355 ± 0.0	1033 ± 0.3
**CH–G**	A549	100 ± 0.1	145.8 ± 0.1	148.3 ± 0.3
	PANC-1	100 ± 0.7	47.9 ± 0.1	59 ± 0.9
**CH–G–HA36**	A549	100 ± 0.4	112.2 ± 0.4	107 ± 0.6
	PANC-1	100 ± 0.8	80.2 ± 0.4	19.9 ± 0.8
**CH–G–HA180**	A549	100 ± 0.4	51.3 ± 0.1	120.4 ± 0.0
	PANC-1	100 ± 0.0	68.5 ± 0.0	117.4 ± 0.2
**CH–G–HA360**	A549	100 ± 0.0	93.6 ± 0.1	141.6 ± 0.1
	PANC-1	100 ± 0.0	54.7 ± 0.0	62.7 ± 0.1

Table S3. Representative SEM-based morphometric analysis of A549 spheroid-like structures observed on CH–G–HA180 scaffolds at day 6.

Parameter	Value
Area	2420 μm²
Feret diameter	90.96 μm
Circularity	0.387
Aspect ratio	1.816
Solidity	0.805

Note: Morphometric parameters were extracted from SEM images using ImageJ 1.54d software (NIH, USA). Values correspond to a representative spheroid-like structure and are intended as supportive morphological descriptors.

Table S4. Broad contextual overview of representative three-dimensional tumor spheroid and scaffold-based culture systems incorporating hyaluronic acid (HA) or related biomaterial components (2012–present).

Study (Year)	Cell Line (s)	Platform Type	HA Component (kDa)	Culture Duration (Days)	Spheroid Outcome	Key Distinction
Lai & Tu (2012)	RCKs	HA-coated 2D surface	35, 360, 1500	1-5	Multicellular spheroid aggregates (76–110 μm) on high MW HA (≥360 kDa)	Established the MW-dependent influence of HA coatings on the self-assembly of spheroids on 2D surfaces [77]
M. J. Ware et al. (2016)	PANC-1 + PSCs	Hanging Drop Co-culture method (Scaffold–free)	N/A	7	High-density PDAC-Stroma Spheroids (PDAC-SS)	Developed a scaffold-free, stromal-rich PDAC model, which focuses on cell-cell interactions in the absence of an exogenous matrix [78]
Monteiro et al. (2021)	PANC-1 + CAFs	Liquid Overlay / Stratified Spheroid (Scaffold–free)	N/A	14	Layered organotypic spheroids with biomarker profiling	Stratified model mimicking in vivo tumor-stroma architecture; demonstrates enhanced chemoresistance [79]
Jiang et al. (2022)	A549	Microfluidic Collagen Hydrogel Chamber Array	N/A	14	Isolated cancer cell proliferation followed by EMT and stromal invasion	Micro-engineered platform to study invasion/EMT within a natural collagen matrix [80]
M. Ermis et al. (2023)	AsPC-1 + CAFs	Methacrylated Hyaluronic Acid/Gelatin (HAMA/GelMA) Hydrogel	Chemically modified HA (Methacrylated HA–HAMA)	14	Compact cancer-CAF spheroids; upregulated EMT/progression markers in stiffer matrices	Tunes matrix stiffness via photopolymerization using methacrylated HA to study its effect on PDAC progression [55]
Demirel et al. (2024)	HepG2	Alginate–Gelatin–HA (Bioprinted)	1.8 MDa (Varied Concentration)	10	Large CD44-mediated spheroids	HA concentration tuning in liver cancer model [68]

Table S4 (continued)

Study (Year))	Cell Line (s)	Platform Type	HA Component (kDa)	Culture Duration (Days)	Spheroid Outcome	Key Distinction
K.G. Pele et al. (2025)	PANC-1 (and Fibroblasts)	Microfluidic Egg White/Gelatin Hydrogel	N/A	14	Grape-like clusters in EW/Gelatin; larger spheroids in Collagen I	Cost-effective EW/Gelatin hydrogel in a microfluidic chip to model PDAC heterogeneity and early angiogenesis [73]
**This Study**	A549 and PANC-1	Freeze-dried Ternary Chitosan–Gelatin–HA Porous Scaffold	36 (LMW), 180 (MMW), 360 (HMW)	6	Compact A549 spheroid-like aggregates and enhanced PANC-1 multicellular organization observed on MMW-HA(CH–G–HA180)	Ternary natural polysaccharide blend (CH–G–HA) introducing an isocompositional HA molecular weight–defined scaffold platform that systematically evaluates the influence of HA MW on scaffold microarchitecture, and scaffold-associated 3D cellular organization

Abbreviations: AsPC-1, human pancreatic adenocarcinoma; CAFs, cancer-associated fibroblasts; PANC-1, human pancreatic carcinoma; PSCs, pancreatic stellate cells; A549, human alveolar adenocarcinoma; HepG2, human hepatocellular carcinoma; RCKs, rabbit corneal keratocytes; N/A, not applicable.

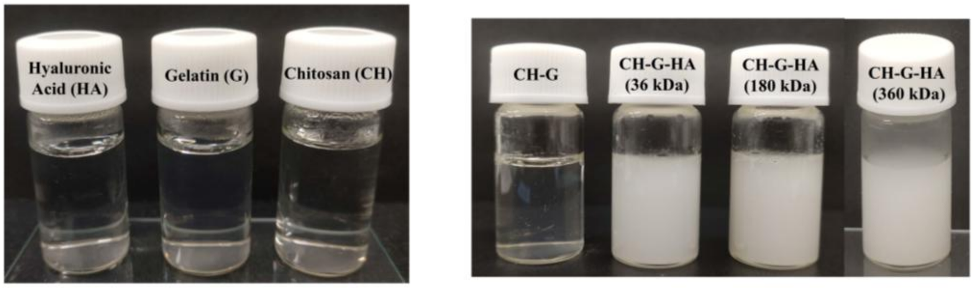

Figure S1. Physical appearance (macroscale) of the polymer solutions.

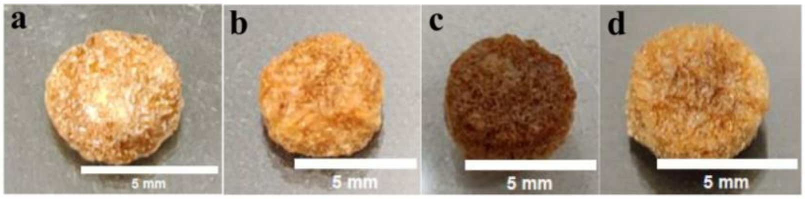

Figure S2. Macroscale morphology of freeze-dried scaffolds: (a) CH–G, (b) CH–G–HA36 (LMW-HA), (c) CH–G–HA180 (MMW-HA), and (d) CH–G–HA360 (HMW-HA).

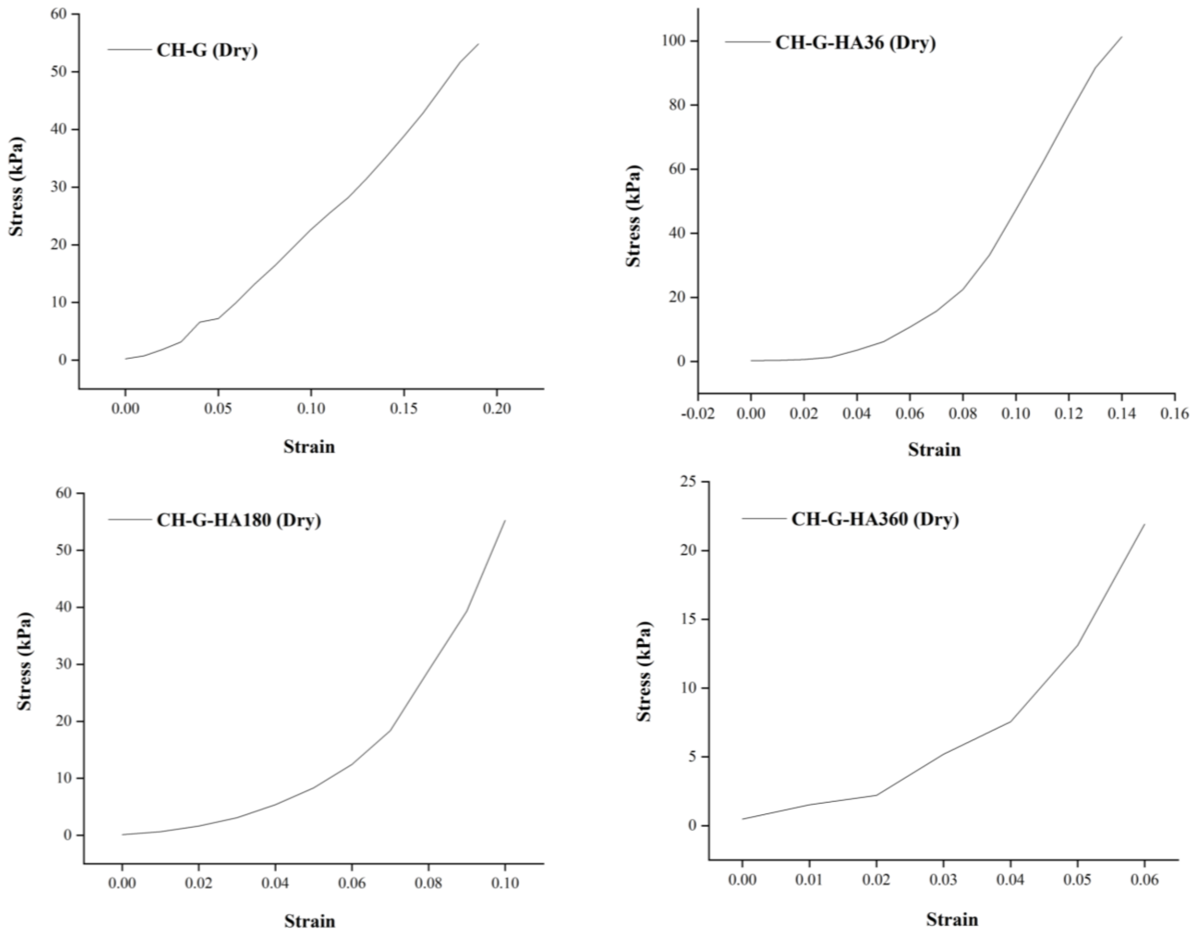

Figure S3. Stress-strain curves for CH–G, CH–G–HA36, CH–G–HA180, and CH–G–HA360 scaffolds under dry experimental conditions, from which the slope in the linear region was determined to be the compressive modulus.

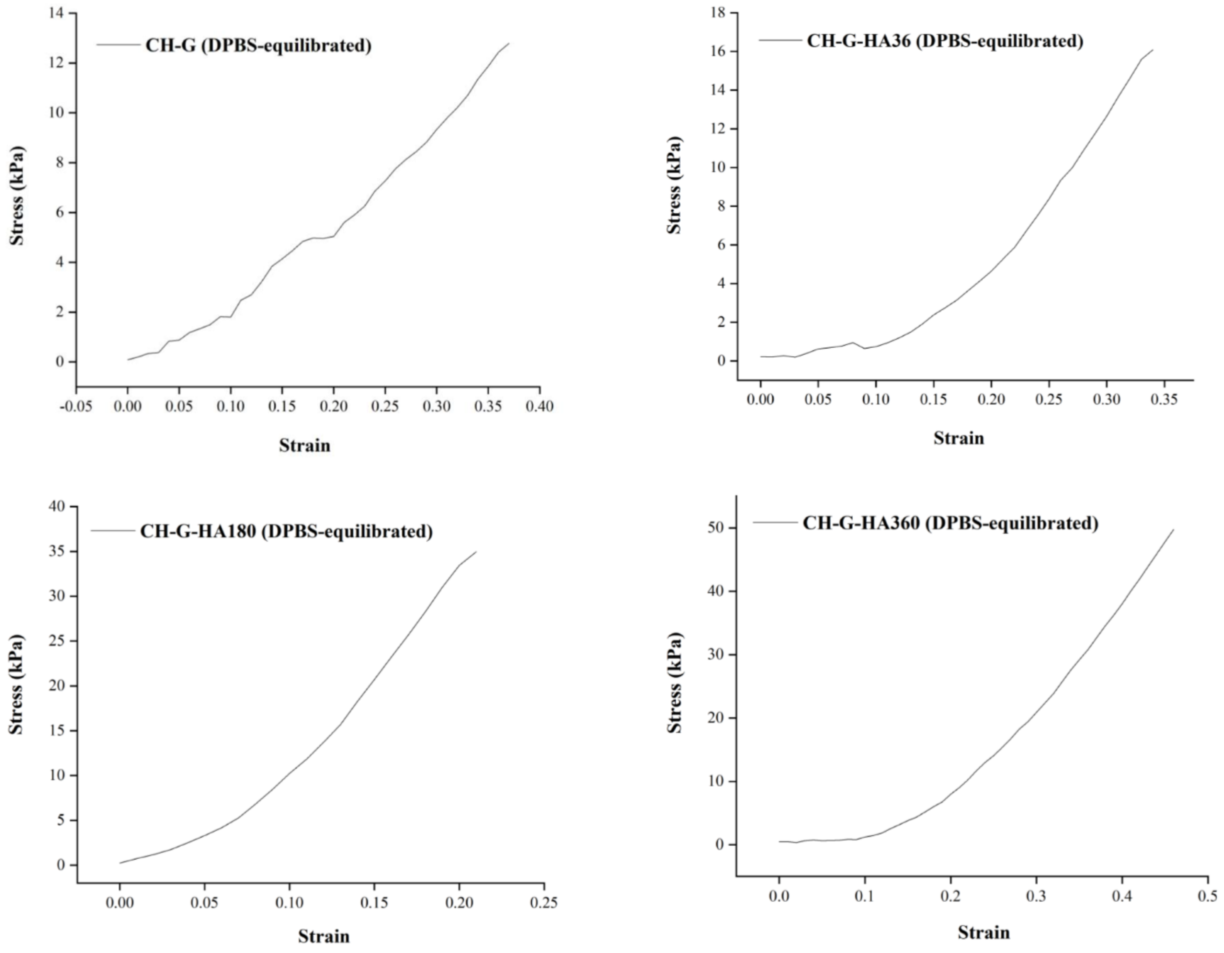

Figure S4. Stress-strain curves for CH–G, CH–G–HA36, CH–G–HA180, and CH–G–HA360 scaffolds under wet (DPBS-equilibrated) experimental conditions, from which the slope in the linear region was used to determine the compressive modulus.

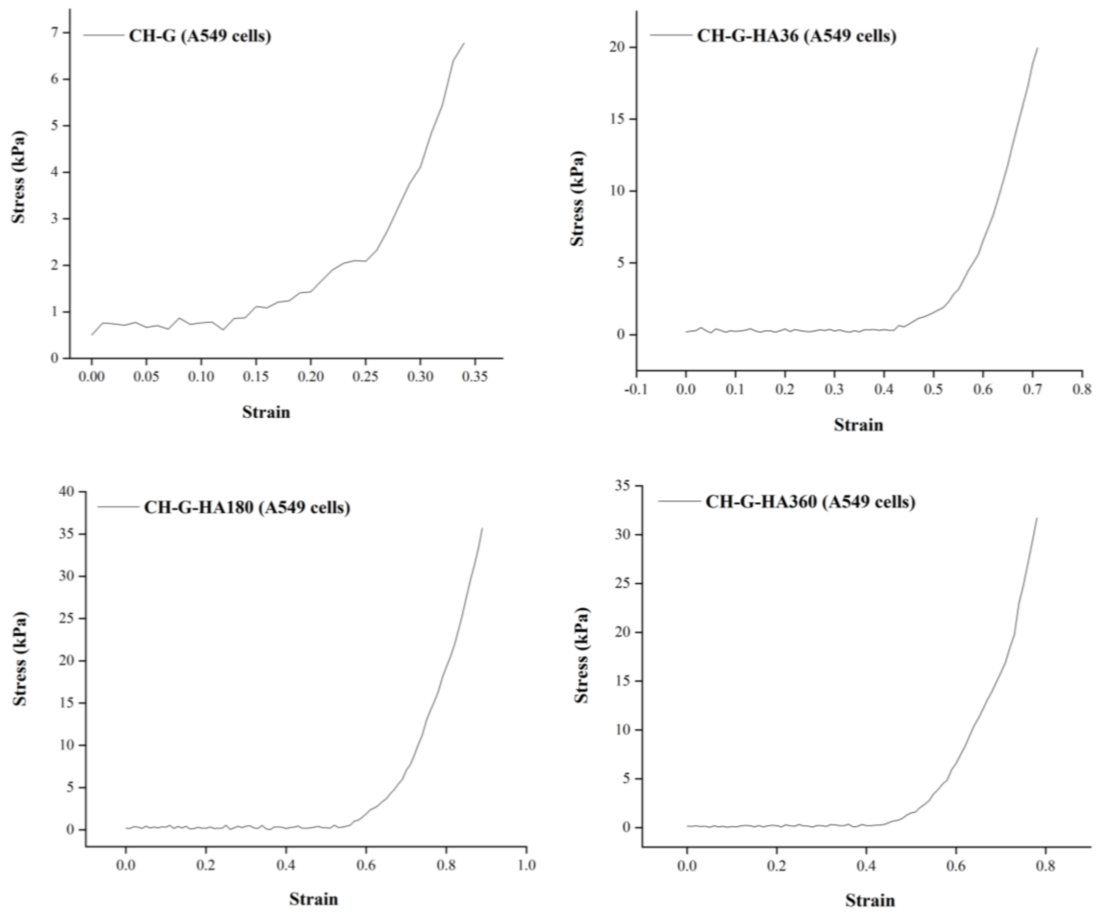

Figure S5. Stress-strain curves for CH–G, CH–G–HA36, CH–G–HA180, and CH–G–HA360 scaffolds after six days of culture with A549 cells, from which the slope in the linear region was used to determine the compressive modulus.

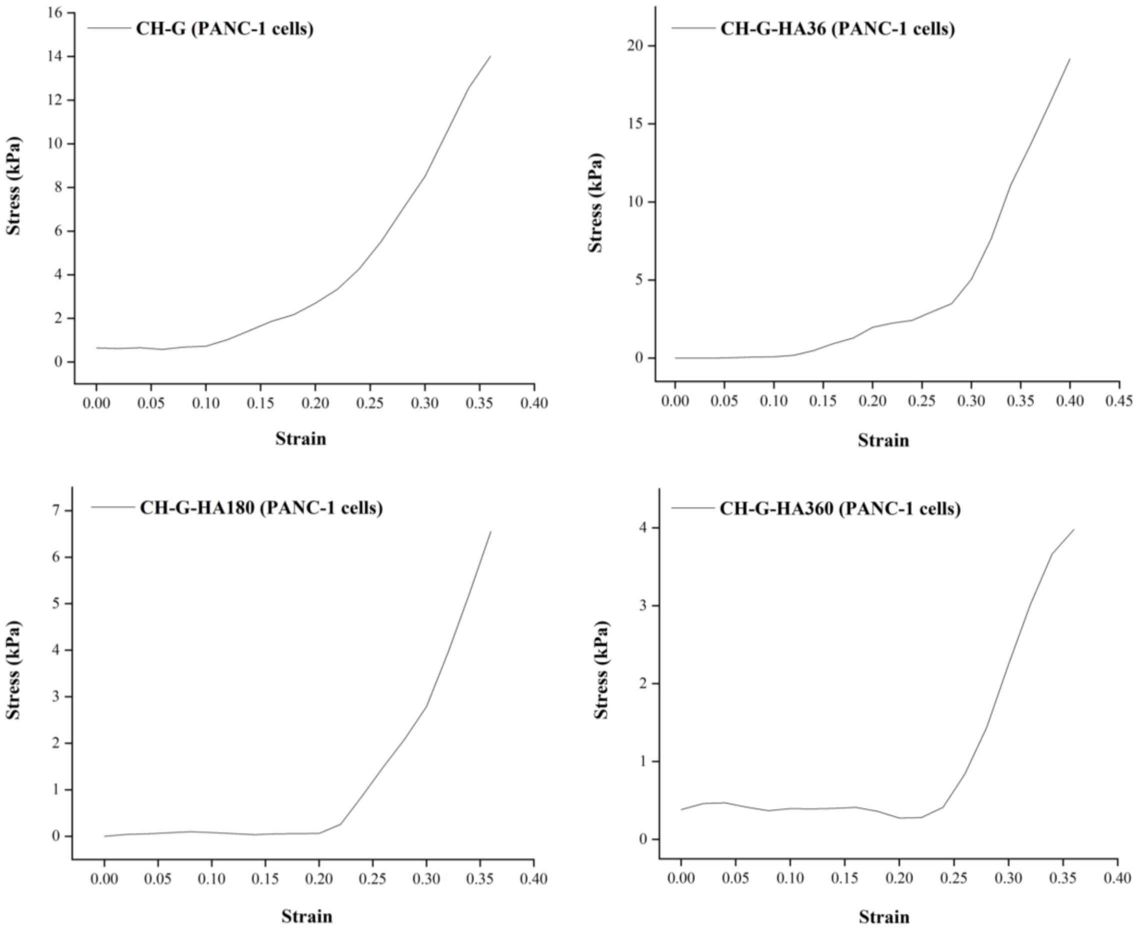

Figure S6. Stress-strain curves for CH–G, CH–G–HA36, CH–G–HA180, and CH–G–HA360 scaffolds after six days of culture with PANC-1 cells, from which the slope in the linear region was used to determine the compressive modulus.
